# Year-round movement of emperor penguin colonies observed from space

**DOI:** 10.1038/s43247-026-03906-0

**Published:** 2026-09-02

**Authors:** Grant J. Macdonald, Stewart S. R. Jamieson, Chris R. Stokes, Peter T. Fretwell, Michelle LaRue, Rose T. N. Foster-Dyer, Stéphanie Jenouvrier

**Affiliations:** 1 Department of Geography, Durham University, Durham, UK; 2 British Antarctic Survey, Cambridge, UK; 3 School of Earth and Environment, University of Canterbury, Christchurch, New Zealand; 4 Biology, Woods Hole Oceanographic Institution, Woods Hole, MA USA

**Keywords:** Ecology, Ecology

## Abstract

Emperor penguins are threatened by climate change and their population is predicted to decline rapidly this century due to changes in Antarctic sea ice. Remote sensing is a key tool for monitoring emperor penguins but analysis has been restricted to spring/summer due to a lack of optical imagery during the polar night. Here we show Sentinel-1 synthetic aperture radar (SAR) can be used to observe the movement of penguin colonies throughout winter. We combine winter SAR imagery with medium-resolution summer optical imagery to track the year-round movement of colonies at Atka Bay, Coulman Island, and Cape Washington, 2017–2024. Although emperor penguins are less mobile than during summer, all three colonies exhibit movement in winter, but which varies between years and colonies. At times, different groups within the colonies synchronously move away from the fast ice edge despite its apparent stability. We demonstrate that SAR is an important tool for understanding emperor penguin behaviour throughout their breeding cycle, and particularly their response to environmental forcing and change during winter.

## Abstract (web summary)

Three of Antarctica’s largest emperor penguin colonies were monitored year-round from 2017–2024, overcoming historical winter observation limitations and demonstrating lower winter than summer movement, according to Sentinel-1 synthetic aperture radar data combined with optical imagery.

## Introduction

Monitoring biological responses to environmental change in Antarctica is challenging because widely used optical remote sensing techniques are rendered obsolete during the dark polar winter. This limitation is especially important for the emperor penguin (*Aptenodytes forsteri*), the only species that breeds during the Antarctic winter. Emperor penguins are ice-dependent seabirds whose breeding success, moulting success, and survival are tightly linked to the stability of land-fast sea ice (fast ice) and the timing of its formation and breakout (e.g. refs. ^1, 2^). As Antarctic sea ice declines, their populations face increasing risk of breeding failure and long-term decline^3^. Although optical satellite imagery has revolutionized understanding of emperor penguin distribution and population trends, its application is restricted to when there is available sunlight. As a result, the movement, persistence and response of colonies to winter weather and changing ice conditions remain largely undocumented. Here, we demonstrate that emperor penguin colonies can be observed during the Antarctic winter by remote sensing using freely available Sentinel-1 synthetic aperture radar (SAR) data. By combining winter SAR and summer optical imagery, we reconstruct continuous seasonal time series of colony positions and movements at three colonies over 8 years.

Emperor penguins breed on fast ice around the continent, close to the margins of glaciers and ice shelves. In order to be successful, they rely on stable fast ice from ~April, when they return from the ocean to the colony, until approximately the end of December, when chicks are ready to fledge. Around mid-May, females lay a single egg and then leave the colony to forage while the male incubates the egg for ~65–75 days^4^. After the egg hatches in July–August, the parents take turns to forage. By September, both adults may make simultaneous foraging trips to feed the chick, meaning the colony is dominated by chicks during late spring^5, 6^. Chicks fledge by January, and it has been suggested that early fast-ice breakout may lead to chicks drowning before their adult feathers have developed^1^, although this is not inevitable given that some observations also suggest chicks may be more resilient to ice loss^7, 8^. Nevertheless, they are potentially threatened by changes in both Southern Ocean sea ice conditions and changes at the margins of the ice sheet, such as major iceberg calving events (e.g. refs. ^9–13^).

Since 2016, Antarctic sea-ice extent and concentration has declined sharply, possibly indicating a new state for sea ice in the Southern Ocean^14–16^. Changes in overall sea-ice extent are not necessarily reflective of changes in fast ice^11, 17–19^, on which emperor penguins breed^10^, and the response of fast ice to evolving climate and ocean conditions is not well resolved. However, in the long term, the loss of large-scale sea ice means that ultimately, fast ice duration and possibly extent are likely to decrease^18^. Furthermore, early fast ice breakout in recent years has led to presumed breeding failure in several regions^11, 12, 15, 20^.

In addition to long-term trends in sea/fast ice, glacier and ice shelf calving may be increasing due to climate change^21–23^. This may also disrupt breeding and foraging because iceberg production can impact sea ice (including fast ice) conditions, access to foraging, and/or disrupt the shelter available to colonies^13, 24, 25^. Indeed, changing habitat conditions are now having a significant impact upon penguin colonies, with a recent continent-wide population analysis estimating that the springtime index of abundance for emperor penguins decreased by 9.6% between 2009 and 2018, and declined in half of the fast ice regions^26^. Furthermore, recent studies have reported a 22% decline in indices of abundance during spring for colonies between 0° and 90°W over the period 2009–2023^27^, and a 32% decline in the Ross Sea region over the period 2020–2024^28^. The future outlook is not positive, with models suggesting that 98% of colonies may be quasi-extinct by 2100 under a high climate warming scenario (Representative Concentration Pathway 8.5) due to sea ice loss, while 61% may still be extinct under a scenario where the Paris Climate Agreement is met, and efforts are made to limit warming to 1.5 °C^3^.

A total of 66 colonies have been identified around the continent, with the majority observed using optical remote sensing^29^. Using medium-resolution (~10–30 m) optical imagery, penguin guano can be identified as a dark stain on the white background of snow/ice, both visually and through automated approaches that use optical and near-infrared spectral values (e.g. refs. ^30, 31^). This has further enabled studies to identify the position of colonies in relation to the sea ice edge or early-breakout events, and to analyse inter-annual changes in location^13, 30–34^. Some very high-resolution optical imagery (VHR; ~<2 m) is available commercially, enabling direct population estimates at some colonies and helping to understand how changing habitat conditions may be impacting the species^26, 27, 32, 35^. In all of the remote sensing studies of emperor penguins, the reliance of optical imagery on sunlight has thus far precluded observations during the winter (between mid-April and late August). This means that, given the harsh environment of the Antarctic polar night, winter observations of the penguins have been restricted to a limited number of field studies at a handful of accessible colonies (e.g. refs. ^4, 6, 36–38^), in addition to data from bird-tagging campaigns (e.g. refs. ^39, 40^) or a remote-controlled observatory^41^. Consequently, relatively little is known about colony movement or behaviour during winter months. This is a key knowledge gap, especially because sea ice decline may impact penguins during the polar night, with late sea ice formation and potentially winter breakout or calving events becoming more common. A report by the German Aerospace Center indicated that emperor penguins are detectable in TerraSAR-X in summer (a high-resolution, tasked, X-band SAR platform)^42^, but they did not present any further analysis.

The aim of this study is to make the first year-round observations of colony positions and, in doing so, generate a satellite time-series of observations of colony location(s) and movement during the polar winter. We combine our winter SAR observations with medium-resolution optical platforms (Landsat 8, 9 and Sentinel-2) to directly determine colony location and movement through a whole breeding season (winter to summer). We explored semi-automated methods to locate and track penguins (see Supplementary Information), which hold clear potential, but found that manual methods validated against optical imagery were currently more reliable, especially in complex sea-ice environments. Our key results are (1) successful detection of emperor penguin colonies in winter on fast ice using Sentinel-1 SAR; (2) detection of guano on ice shelves in summer using Sentinel-1 SAR; (3) demonstrated year-round tracking of colony movement combining medium-resolution optical and SAR remote sensing, and (4) seasonal tracking of colonies and multiple sub-groups reveals key colony dynamics, such as timing of movement onto the ice shelf at Atka Bay, and behaviours indicative of responses to meteorological drivers.

## Results

**Fig. 1:**
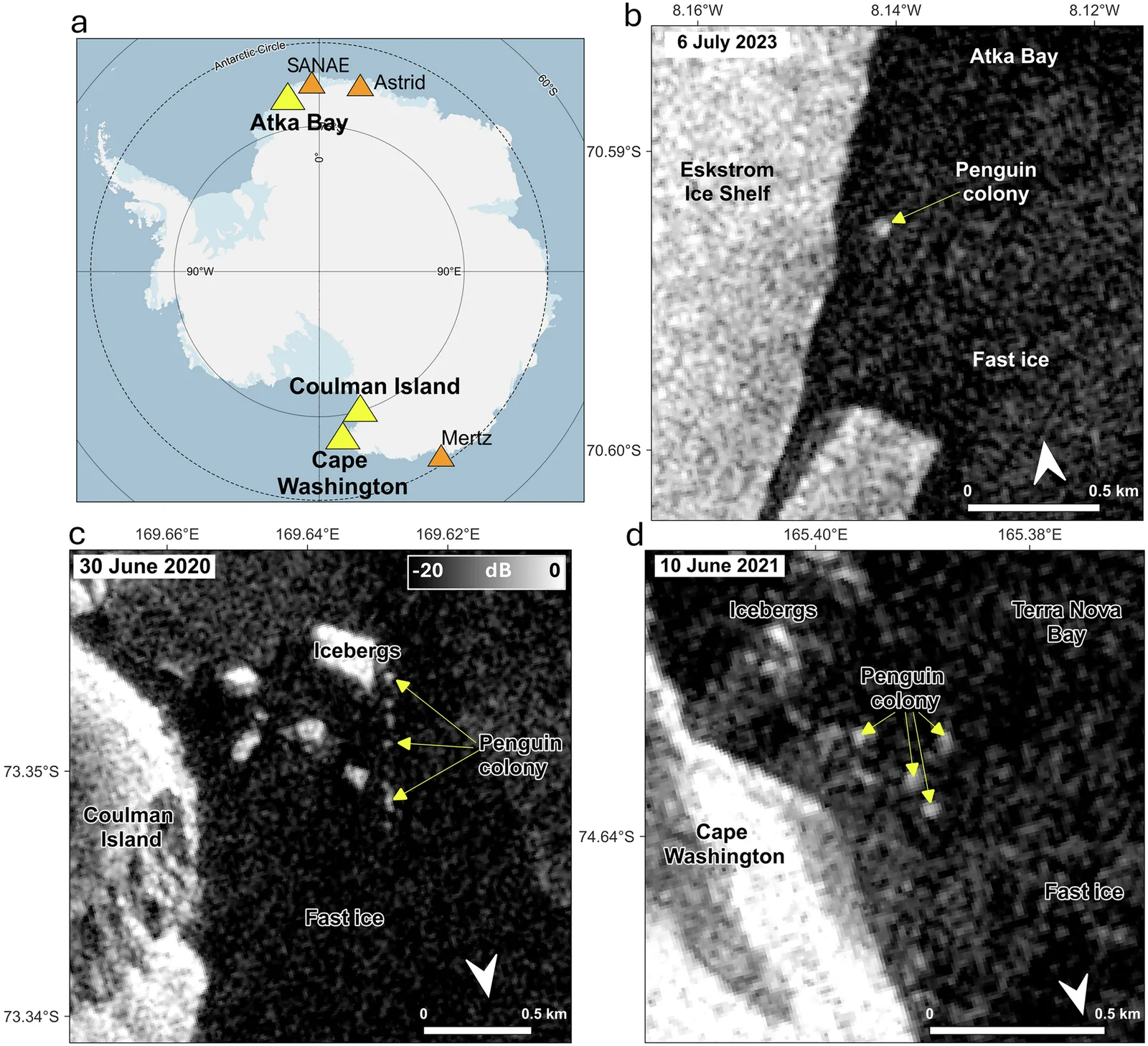
The locations of study sites and examples of penguin colonies visible in Sentinel-1 SAR. **a** The locations of the three primary study sites are in yellow and additional sites where we observed colonies in SAR are in orange (the basemap is from Quantarctica^63^); the colonies visible in Sentinel-1 at **b** Atka Bay, **c** Coulman Island and **d** Cape Washington.

We map year-round movement of the Atka Bay (~8.2°W, 70.6°S), Coulman Island (~169.6°E, 73.4°S) and Cape Washington (~165.4°E, 74.6°S) colonies, but also provide examples of additional colonies that are visible in SAR at SANAE, Astrid and Mertz (Figs. 1 and S1). As two of the largest emperor penguin colonies in existence^32^, the Coulman Island and Cape Washington locations are important for the global population, while Atka Bay is also large and of particular interest due to its movement from the fast ice onto the neighbouring ice shelf^41, 43^. For these reasons, and their location near research stations, the colonies already constitute some of the most-studied and are key for understanding the species more broadly. The most recent springtime counts indicate that the Atka Bay, Coulman Island and Cape Washington colonies had adult populations of 9657^32^, 19,822^44^ and 12,178^44^, respectively. We manually track the colonies using Sentinel-1 SAR through the winter (~April/May–September) and optical imagery (Landsat 8 and 9 and Sentinel-2) in the spring/summer (September–January) for the years 2017–2024 (see 'Methods'), which often enables us to record sub-weekly changes in location.

### Observation of winter penguin colonies on fast ice using SAR

We found emperor penguin colonies were visible in Sentinel-1 SAR interferometric-wide (IW) swath mode (10 m resolution) horizontal-horizontal (HH) images during winter on fast ice as relatively bright (high backscatter) pixels against a background of darker fast ice (e.g. Fig. 1b–d). We also found them in some extra-wide (EW) mode (40 m resolution) and horizontal-vertical (HV) channel images (see 'Methods'), but focus on IW HH images because IW mode has the highest resolution and the HH channel is more often available at the sites. We successfully located the colonies in all years of the study period (2017–2024) at Atka Bay (Fig. 3 and Video S1), Coulman Island (Fig. 4 and Video S2) and Cape Washington (Fig. 5 and Video S3). Additionally, we note that the colonies were visible at least in some years at SANAE, Mertz and Astrid (Fig. S1). In some cases, the colony was very distinct, while in other cases rough and older ice features, which have a higher backscatter than smooth winter first-year ice, meant that much of the ice had a similar backscatter to the colony. In those cases, the colony can still be observed due to the movement of the colony across successive images, in contrast to the stationary fast ice directly around them. We note that there were instances where high backscatter from the rough fast ice conditions made it very challenging and, in some cases, impossible to locate the colony, such as at another colony we tested, Snow Hill (Fig. S2).

**Fig. 2:**
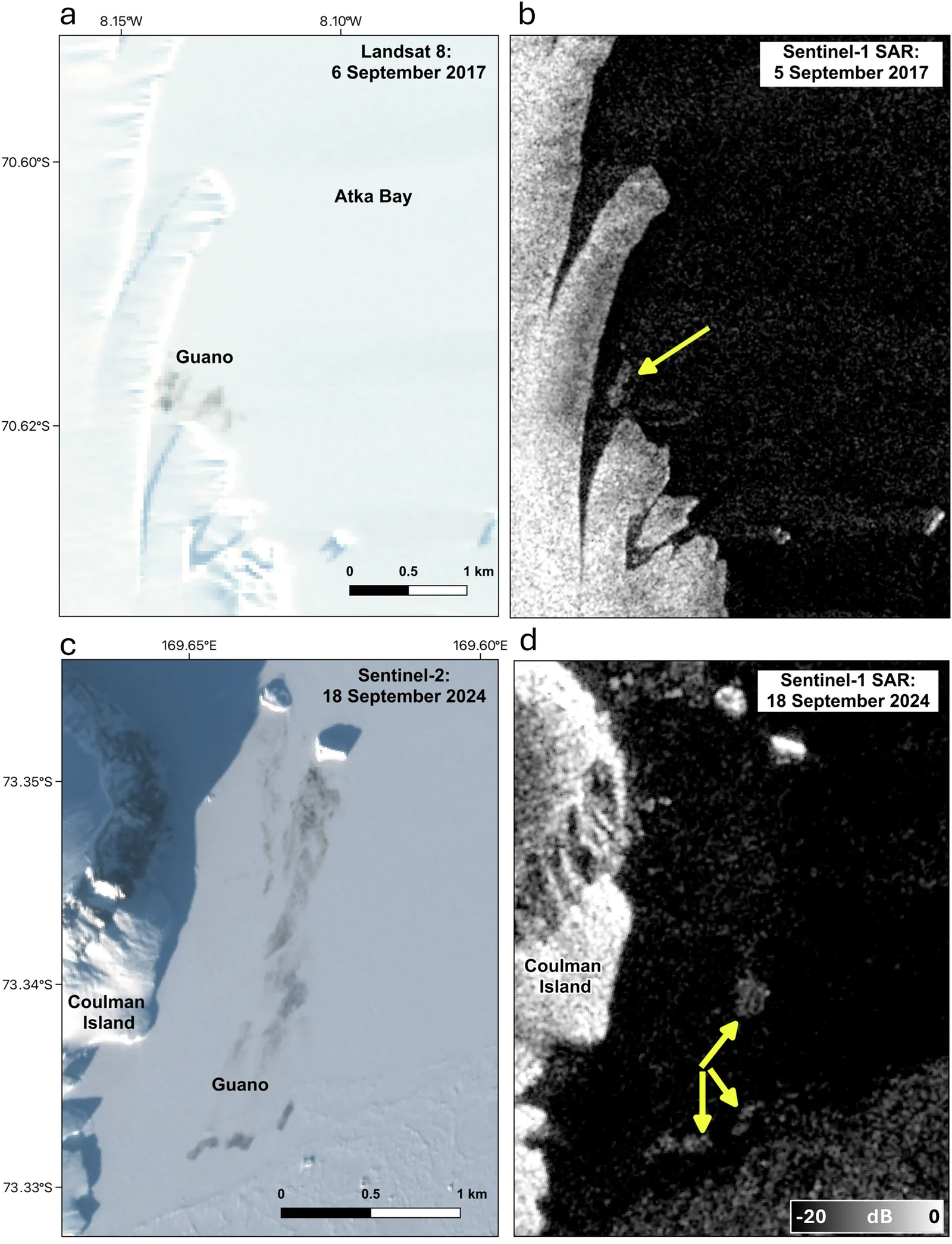
Penguin guano and colonies in (near-)simultaneous optical and SAR imagery. **a** A Landsat 8 optical image and **b** Sentinel-1 SAR image at the same location in Atka Bay 1 day apart; **c** a Sentinel-2 optical image and **d** Sentinel-1 SAR image at the same location at Coulman Island on the same day. Yellow arrows point to bright areas showing the colony, where guano can also be seen in the same area in the optical imagery. An additional example at the SANAE colony in late August is shown in Fig. S3.

Coincident or near-coincident optical and SAR images in late August and September, when light returns, provided additional validation that we were correctly identifying penguin colonies in the SAR imagery. Specifically, in near-coincident optical/SAR images, bright patches in the SAR were visible in the same locations as darker areas within the guano-stained ice (Figs. 2 and S3), with much of the rest of the guano presumably being devoid of penguins at the time. Optical imagery from earlier winter months does not exist for validation, but these August/September images serve as the best available for the winter period, as the fast ice conditions and the state of the penguin colony are broadly representative of this winter period, and the presence/absence of sunlight does not impact the SAR signal. Unlike guano in optical imagery, the SAR signal of a colony on winter fast ice did not consistently expand but instead shifted location between images. This indicates that the enhanced backscatter was generated primarily by the penguins themselves rather than by a trail of guano deposits and demonstrates that the SAR signal may be useful for more closely tracking colony movement (e.g. distinguishing it from surrounding guano staining), even when optical imagery is available.

**Fig. 3:**
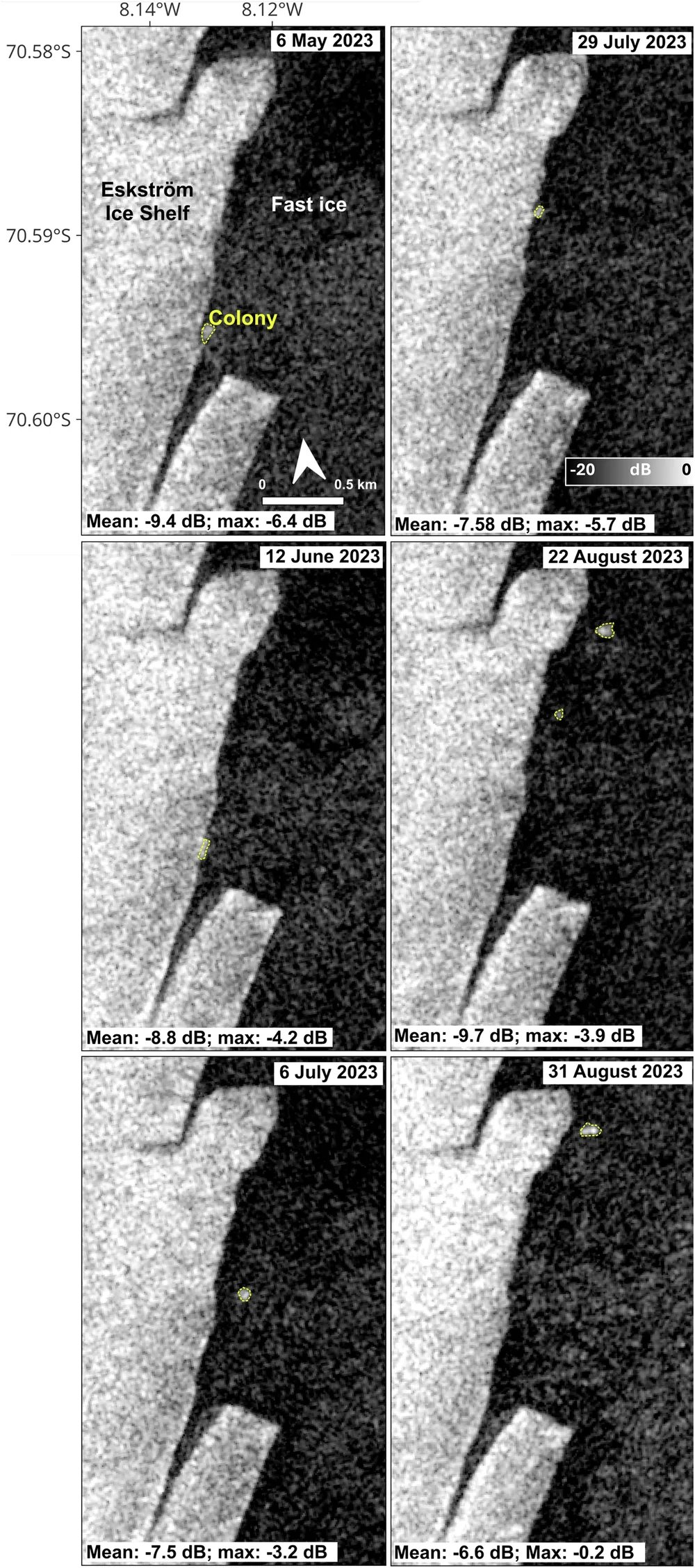
The Atka colony visible on fast ice throughout winter in 2023 in Sentinel-1 IW HH imagery. The colony pixels are within the yellow dashed line boundary, within which the mean and maximum backscatters were calculated. All Sentinel-1 IW HH images for each winter 2017–2024 at Atka are produced as timelapses in Video S1.

**Fig. 4:**
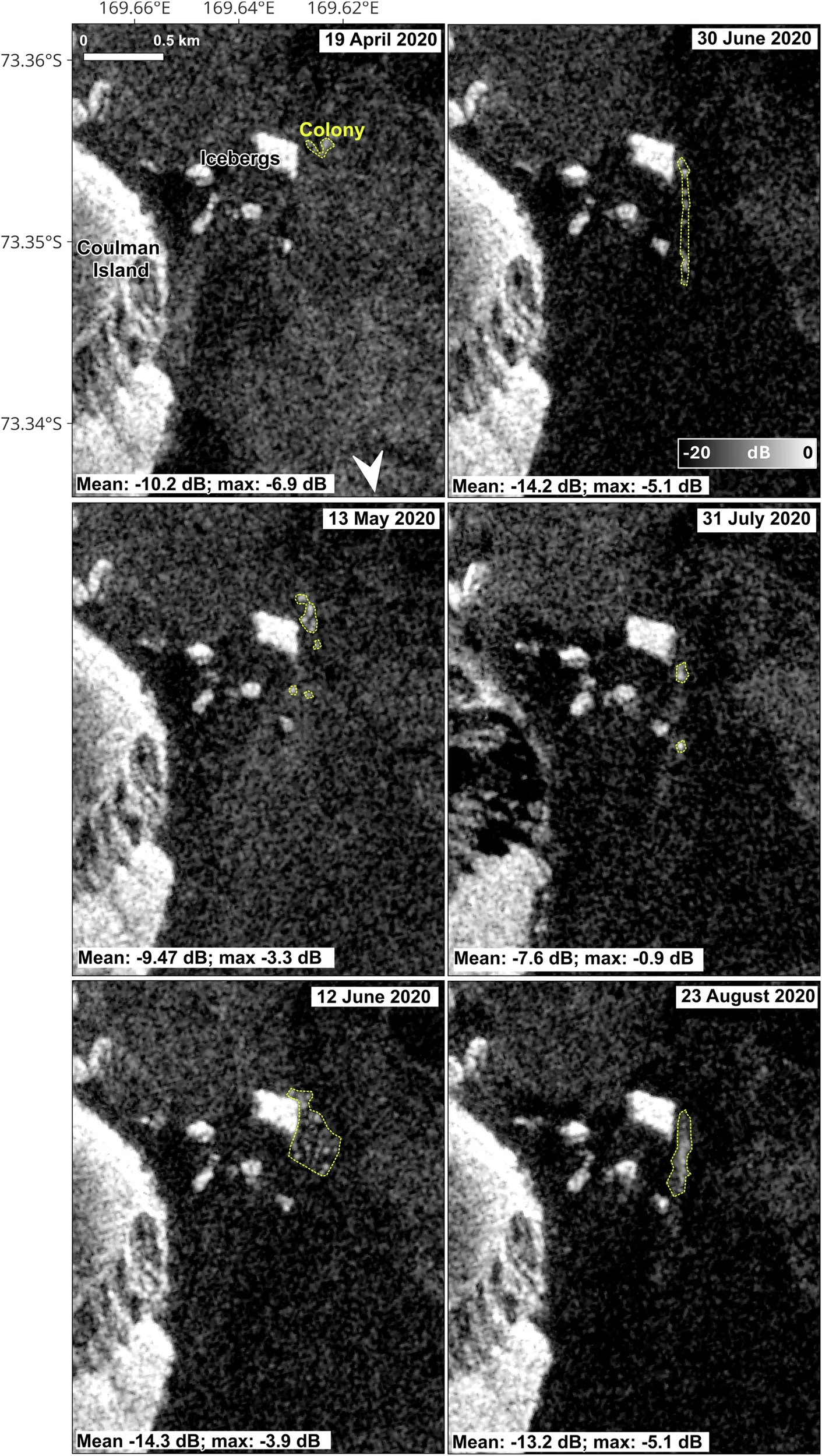
The Coulman Island colony visible on fast ice throughout winter in 2020 in Sentinel-1 IW HH imagery. The colony pixels are within the yellow dashed line boundary, within which the mean and maximum backscatters were calculated. At times the colony is spread out into many small groups, and non-colony pixels are also within the yellow boundary. All Sentinel-1 IW HH images for each winter 2017–2024 at Coulman Island are provided as timelapses in Video S2.

**Fig. 5:**
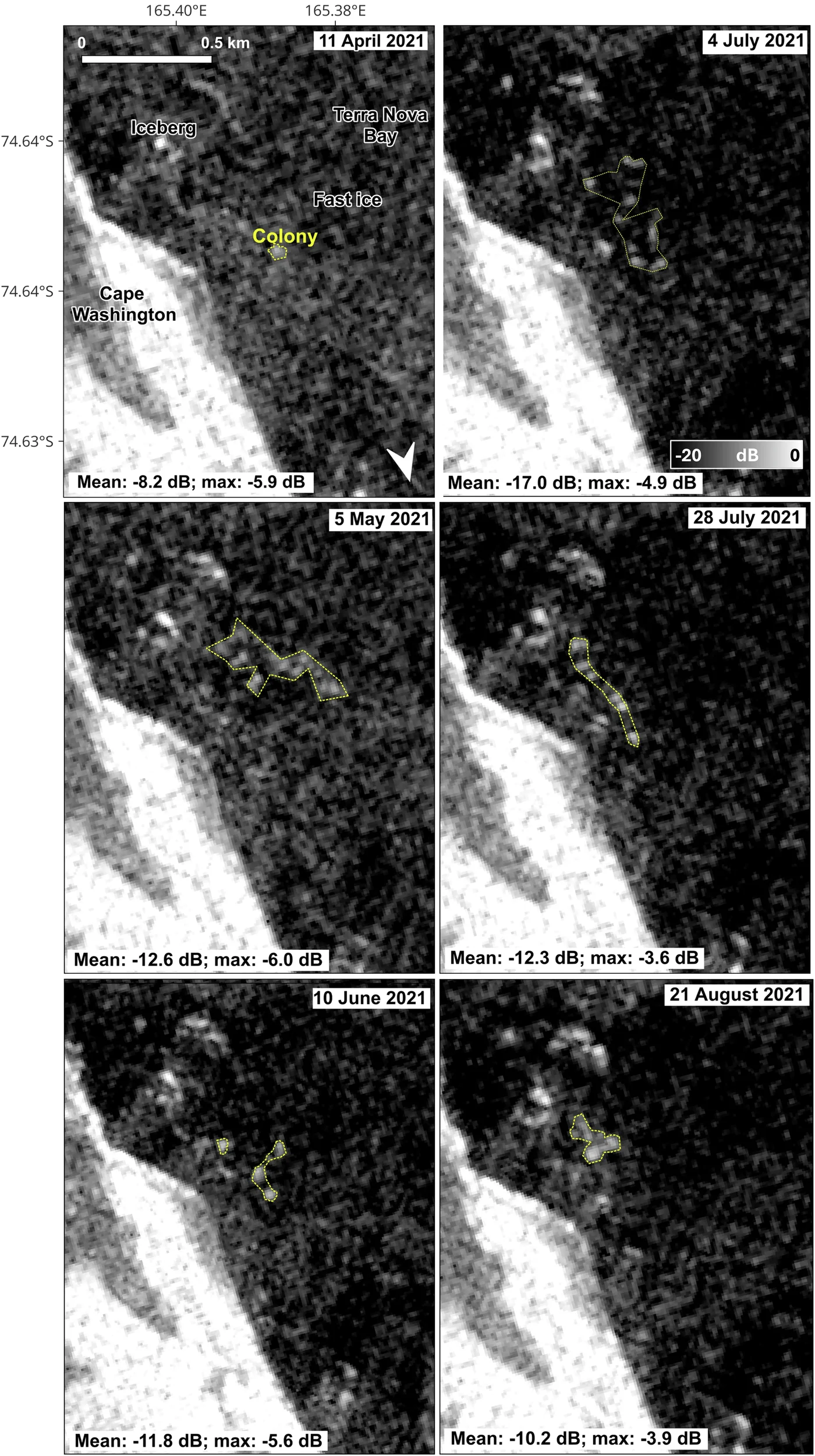
The Cape Washington colony visible on fast ice throughout winter in 2021 in Sentinel-1 IW HH imagery. The colony pixels are within the yellow dashed line boundary, within which the mean and maximum backscatters were calculated. At times the colony is spread out into many small groups, and non-colony pixels are also within the yellow boundary. All Sentinel-1 IW HH images for each winter 2017–2024 at Cape Washington are produced as timelapses in Video S3.

The colonies were sometimes difficult to identify in early winter (April), partly due to the fast ice typically having higher backscatter, while the colony was also more diffuse, smaller, and therefore characterised by lower backscatter and was less coherent. Consequently, the exact timing of colony-return was difficult to confirm from the SAR imagery. However, once a colony first became visible between mid-late April and early May (June in one case at Coulman Island in 2021), we were able to observe it throughout the rest of the winter until optical images became available (August/September). Sometimes the colony was visible in one consolidated group of pixels (e.g. at Atka Bay in Fig. 3) or often as a cluster of several groups that moved together over multiple images (e.g. at Coulman Island and Cape Washington in Figs. 4 and 5). The form of colonies changed through the season, becoming more or less closely grouped, and splitting and re-joining (e.g. Figs. 4 and 5 at Coulman Island and Cape Washington). Typically, the colony at Atka Bay was more clearly identifiable than at the other two colonies, exhibiting a denser and more coherent grouping of penguins, whereas the other two colonies were more diffuse.

### Identification of guano on ice shelves using SAR

**Fig. 6:**
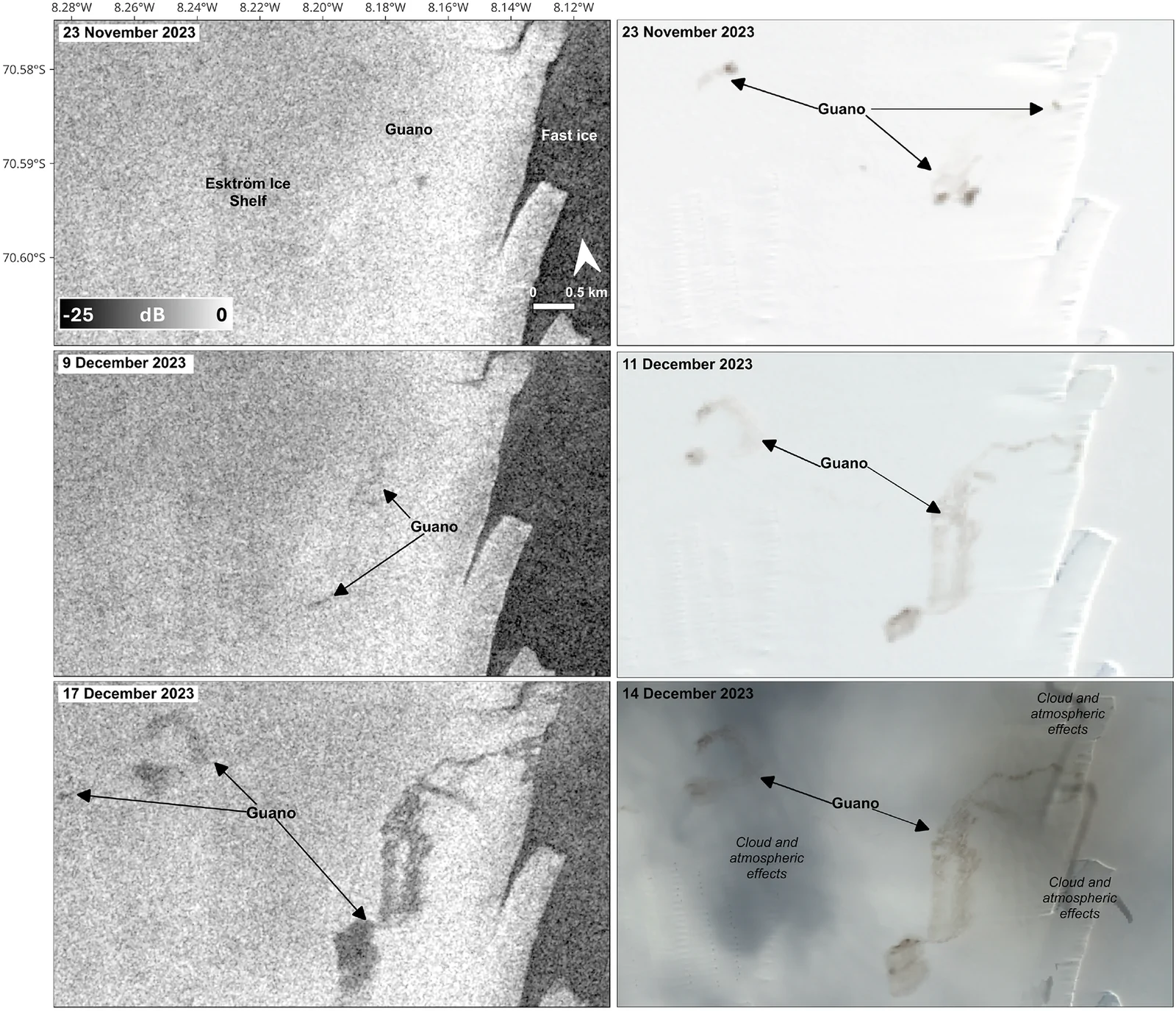
Sentinel-1 SAR images and Landsat 8 optical images of the ice shelf onto which the Atka Bay colony moves, over approximately the same time period in the summer of 2023. A guano signal is visible in the SAR images, as well as in the optical. However, while extensive guano is visible in all optical images, it is only extensively visible in the final SAR image.

At Atka Bay, the colony moved onto the adjacent ice shelf (composed of glacier ice) at the end of the winter in seven of the 8 years, at which point the colony was no longer visible in Sentinel-1 against the bright backscatter of the ice shelf. However, later in the summer, a distinct low backscatter region on the ice shelf became visible in the SAR imagery that corresponded to the location of the guano that was visible in the optical imagery (Fig. 6). Notably, this low backscatter signal was not visible throughout the whole summer, even if guano was present and visible in the optical imagery. Additionally, the dark region did not spread spatially, as it would if it represented ongoing colony movement. Instead, the whole guano-stained part of the ice shelf gradually became visible in SAR imagery later in the summer. This suggests that while the high backscatter regions on the fast ice represent direct observations of the actual penguin colony, the late-summer, low-backscatter region on the shelf represents the area covered by guano or other impurities associated with a breeding site, only some of which will contain penguins at that time. We similarly identified guano on the ice shelf at SANAE using SAR imagery, as confirmed by optical data (Fig. S4).

### Year-round colony movement

**Fig. 7:**
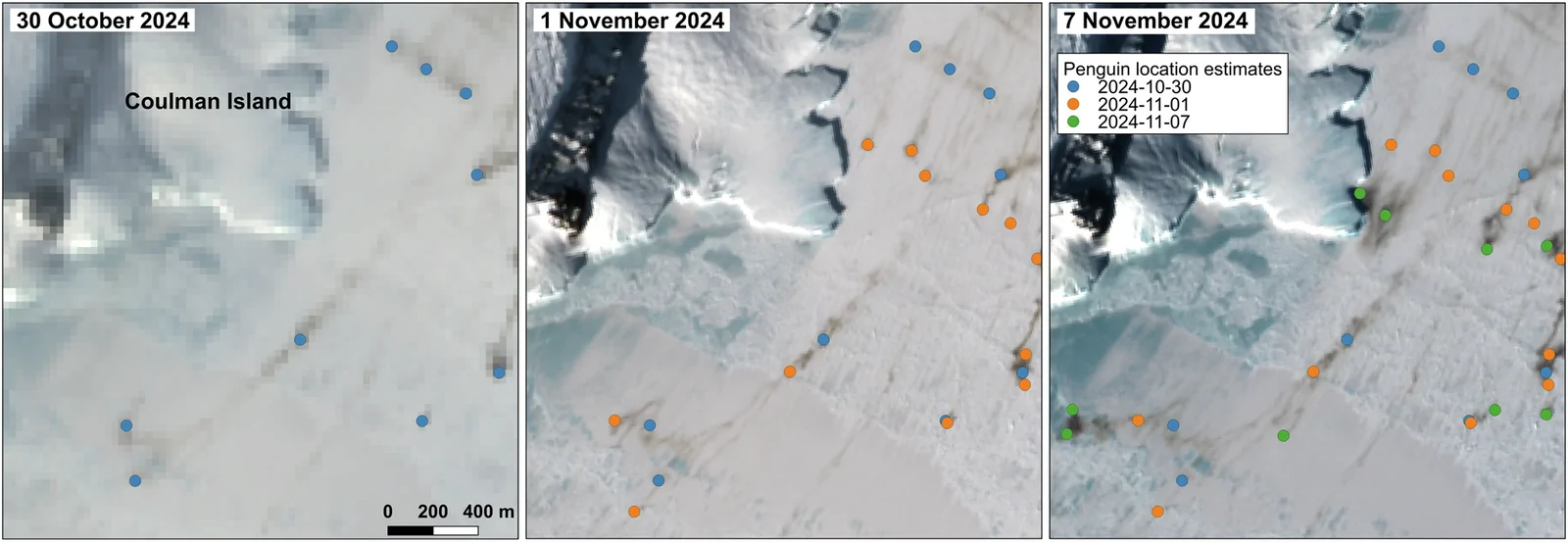
Penguin tracking from guano at the Coulman colony in the summer of 2024. Estimated location of penguins in each of a series of consecutive Landsat 8 and Sentinel-2 images, based on visual tracking of guano. Coloured dots show the estimated location of groups within the colony on each of the three dates.

The visibility of colonies in Sentinel-1 during winter, and in multiple optical instruments during summer, enabled us to manually track their movement throughout the breeding season at a high temporal resolution (e.g. Fig. 7). Although there are limitations and challenges to such tracking, primarily due to spatial resolution (see 'Methods'), we captured the year-round movement of colonies at a sub-weekly timescale over 8 years, including of sub-groups within a colony. As noted above, a semi-automated approach was tested but generated numerous false positives, which then needed to be manually edited, meaning the approach did not perform well in more complex sea-ice environments (see 'Methods' and Supplementary Materials).

**Fig. 8:**
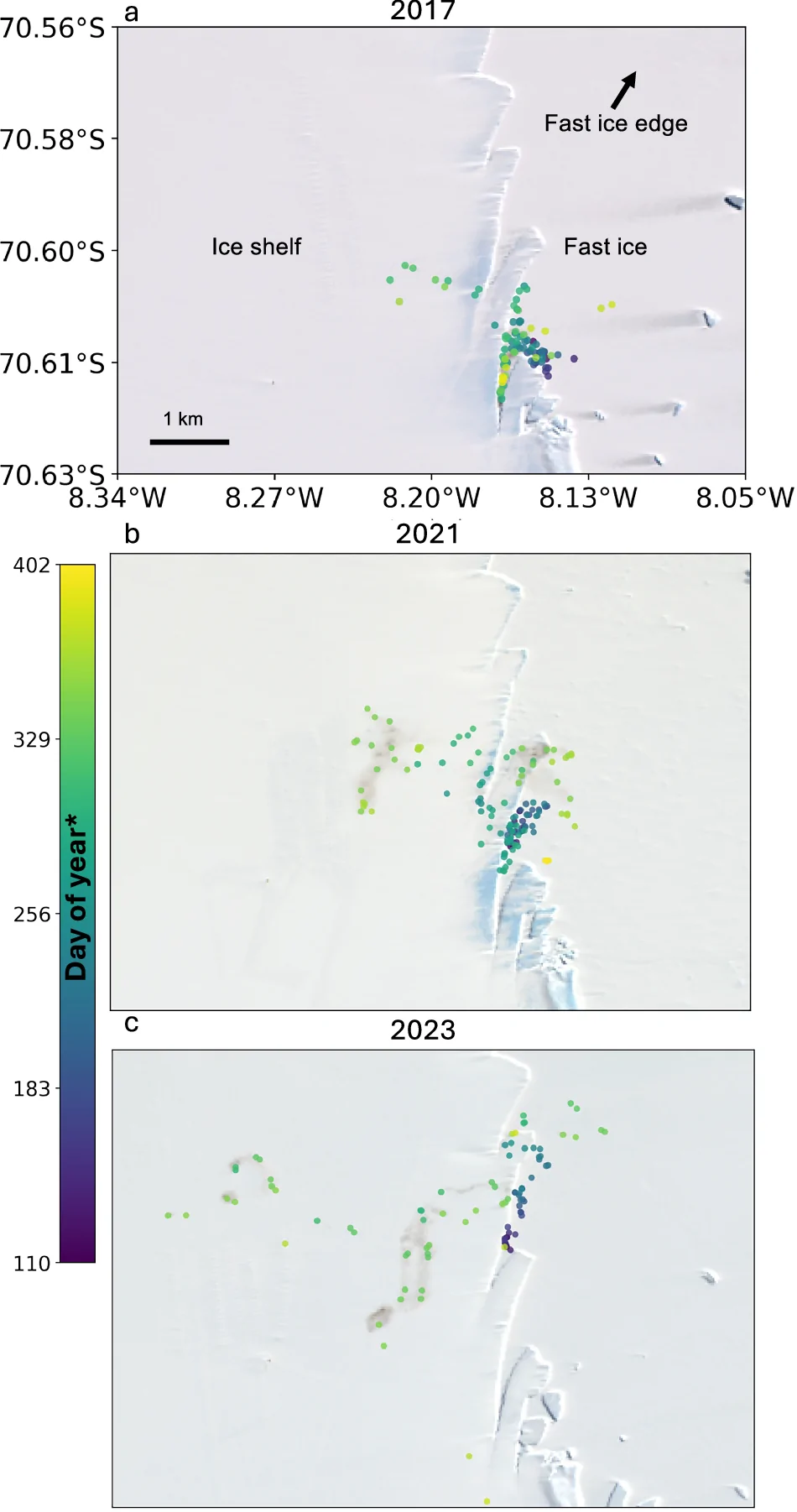
Examples of the Atka Bay colony’s year-round movement. Movement is shown in **a** 2017 (background image: 30 November 2017), **b** 2021 (background image: 13 December 2021), and **c** 2023 (background image: 11 December 2023). Dot colour represents the day of year (doy) of colony location; a doy over 365 signifies it is after 1 January of the following year. Note many dates have multiple dots due to splitting of the colony. Tracking results for all eight study years for Atka Bay are shown as animations in Video S4.

**Fig. 9:**
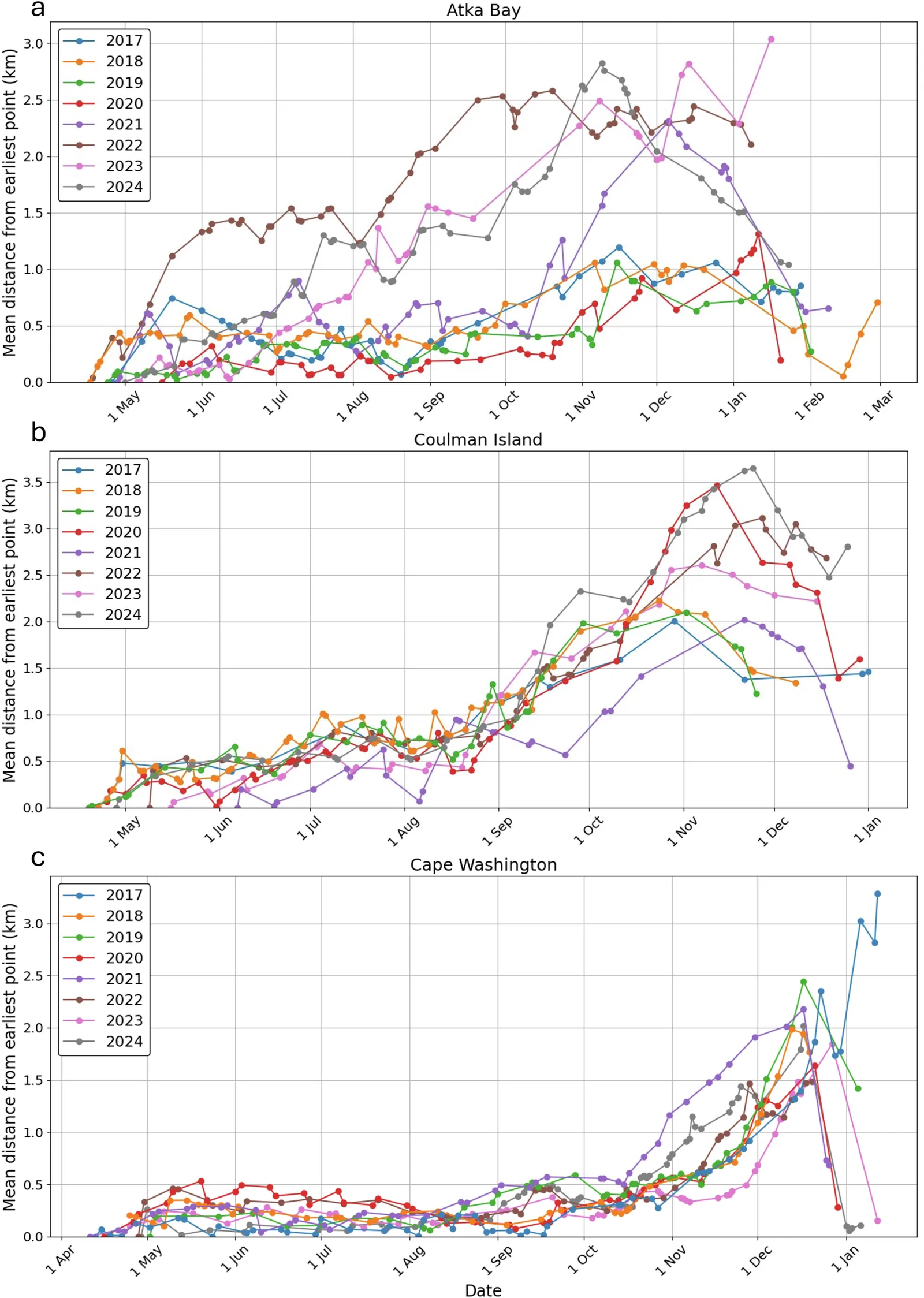
Mean distance for all subgroups from their first location of the seasonat the three colonies, 2017-2024. Mean distance at the **a** Atka Bay, **b** Coulman Island, and **c** Cape Washington colonies for 2017–2024. The mean distance of all subgroups of the colony on each day is calculated from the earliest location of the colony in that year. In the instance (2024) where there are two separate groups at Atka Bay throughout winter in different embayments, the distance is calculated for the southern of the two groups.

At Atka Bay, the colony was first visible in April or May in a bay of fast ice adjacent to the ice shelf (Fig. 8 and Video S4). From 2017 to 2021, they spent winter on the fast ice within the same small shelf-margin embayment, which advects northwards due to glacier flow throughout the study period. Associated with this advection, the colony generally moved northwards over the study period, with the earliest winter observations for each of the 8 years spread over a maximum of 2.30 km. In 2022, they moved northwards to an adjacent small embayment along the ice shelf margin in late May, which was the greatest winter movement in this year (>1.5 km by July; Fig. 9a). In 2023, they spent all winter in that northern embayment and in 2024 the colony split into two separate groups, with one in each embayment. During winter, the colony typically moved less than it did later in summer; and in four of the eight winters the colony was within 0.50 km of its earliest location by the end of August (Fig. 9a). In all but one year (2018) the colony moved onto the ice shelf for part of the year. We observed this shift onto the ice shelf by 3rd September at the earliest (2022), and 28th October at the latest (2019), and it occurred by late September in all other years. Notably, the movement of the colony onto the ice shelf occurred despite the fast ice being apparently stable. Through the summer, the colony was typically more dynamic, moving further into the ice shelf interior and away from the fast ice edge, extending to over 2.00 km from its original location in four of the 8 years. During this time, the colony split into numerous groups (up to eight) that are discernible in the optical images and spread out across the ice shelf and fast ice, at times moving in the same direction as each other, and at others independently. Some groups travelled further than others, with the maximum distance travelled by groups (Fig. S5A) significantly surpassing the mean (Fig. 9a), and with one group travelling 5.57 km in 2023. Groups were observed returning from the ice shelf to the fast ice between October and January. Although all surviving penguins presumably returned to the fast ice/ocean eventually, only some groups were observed returning from the ice shelf to the fast ice before becoming undetectable.

**Fig. 10:**
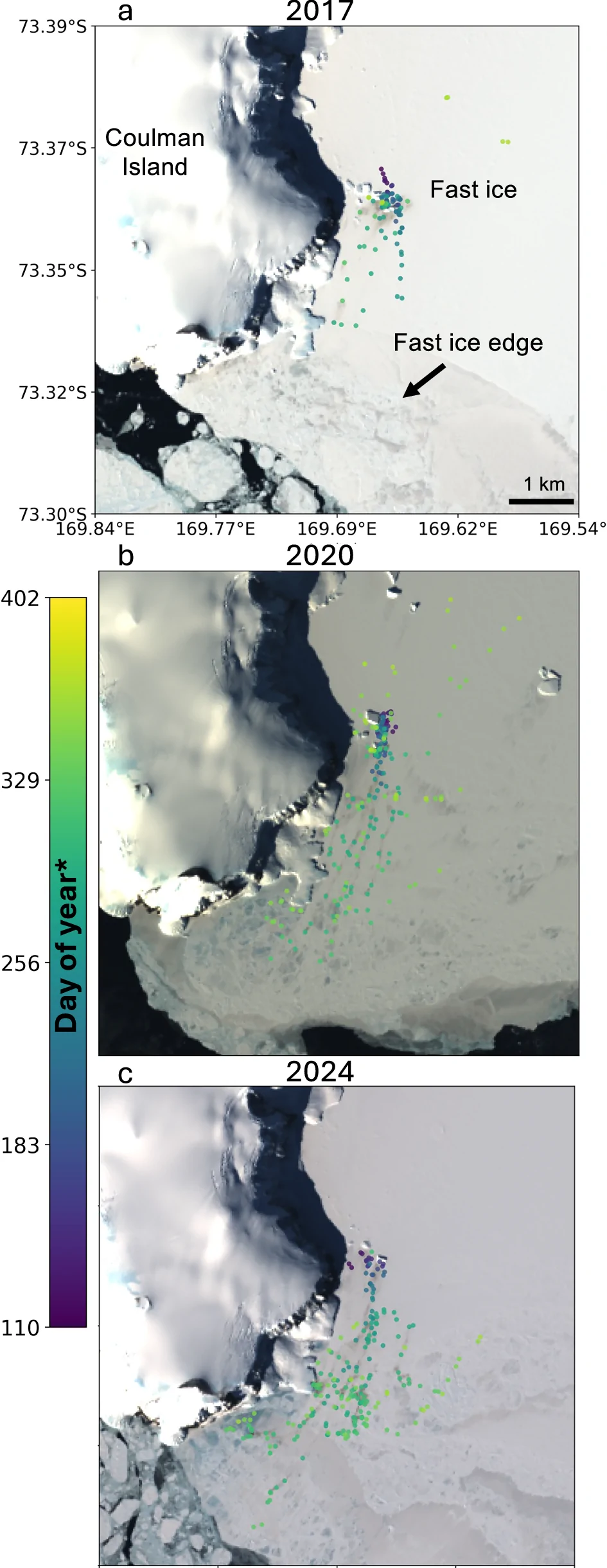
Examples of the Coulman Island colony’s year-round movement. Movement is shown in **a** 2017 (background image: 21 November 2017), **b** 2020 (background image: 28 October 2020) and **c** 2024 (background image: 8 November 2024). Dot colour represents the doy of colony location; a doy over 365 signifies it is after 1 January of the following year. Note many dates have multiple dots due to splitting of the colony. Tracking results for all eight study years for Coulman Island are shown as animations in Video S5, and for Cape Washington in Video S6.

At Coulman Island, the colony returned to the same approximate location each winter (the first winter observations from each of the 8 years were spread over 1.10 km), on fast ice adjacent to the island (Fig. 10 and Video S5). A cluster of icebergs was embedded in the fast ice in approximately the same location each year, around which the colony was located in 1–3 main groups each winter. Every year, the colony moved around this area for most of winter, typically not moving more than 1 km from its earliest winter location before late August or September (Fig. 9b). Thereafter, and throughout summer, the colony split into multiple visible groups (up to 19). Typically, these initially moved north towards the ice edge and, by late October or early November, they reached a mean of between 2 and 3.5 km from their earliest location depending on the year (Fig. 9b), before turning away from the ice edge again. Such a turnaround often happened simultaneously across numerous groups dispersed over several kilometres and occurred without any visible change in ice conditions (e.g. see 21 November–7 December 2024 in Video S5h). Although most groups were highly mobile (one group reaching 5.73 km away in 2025; Fig. S5b), some penguins also remained at the initial winter location around the icebergs, evident from the renewal of staining. By the beginning of January, guano staining typically became very faint and did not evolve spatially, suggesting the vast majority of penguins were no longer present at Coulman Island.

The Cape Washington colony was consistently situated on fast ice near the southern tip of the cape, where icebergs also embedded at the beginning of every winter. The colony’s inter-annual location was the most consistent of all colonies, with the first winter observations of the 8 years spread over just 0.6 km. The colony consisted of 1–3 main groups during winter, remaining within 0.5 km of the earliest location in almost all cases between April and August. Between October and November, the colony became significantly more mobile, forming as many as 14 groups and moving a mean of between 1.50 and 2.50 km away from the initial winter location by mid-December (Fig. 9c), although (as with Coulman Island) some penguins remained at their original location. As at the other colonies, some groups travelled further than others, with the maximum recorded distance of a group being 5.64 km in 2017 (Fig. S5c). Penguin movement was often away from the ice edge (Video S6) and, similar to the other colonies, groups often simultaneously moved in the same direction as each other (including simultaneous changes in direction) and not in response to any visible changes in ice conditions. By late December or early January, most groups appeared to have left the colony, apart from some that remained at the initial winter location.

## Discussion

### Observation of emperor penguin colonies in winter using Sentinel-1

We show that emperor penguins are observable in Sentinel-1 SAR and present year-round remote sensing observations of colony movement. Sentinel-1 and other C-band synthetic aperture radar (SAR) instruments are sensitive to a range of sea ice characteristics, such as roughness, age and meltwater content and have been used extensively to study a variety of sea ice conditions (e.g. refs. ^45–47^). It has also been used to analyse aspects of glaciers and ice shelves, such as the presence of meltwater, including supraglacial and englacial lakes (e.g. refs. ^48–50^). However, Sentinel-1 has not been used to analyse penguins, even though, unlike some high-resolution SAR instruments, Sentinel-1 routinely collects imagery around the Antarctic and does not need to be requested.

Significantly, and unlike medium-resolution optical imagery, the winter SAR signal from the colony on fast ice appears to be from the colony itself rather than guano, given that the signal moves with the colony rather than leaving a residual trail of where the colony has been. Without separating the biogenic contribution to the radar signal from the background ice, for example, via polarimetric decomposition, we cannot determine exactly why the colony exhibits a higher backscatter than the fast ice. However, we hypothesise that it is likely to be at least partly due to penguins standing tall (>1 m) above the fast ice and acting as a surface of variable elevation, thereby introducing additional roughness beyond the background levels from the sea ice. Rough ice and ice features have been shown to cause higher C-band SAR backscatter, including in Sentinel-1, because rough features on the order of, or exceeding, the wavelength of the signal cause increased surface scattering towards the instrument (e.g. refs. ^47, 51^). In addition to roughness, the dielectric properties of the birds’ bodies and plumage may impact scattering towards the instrument.

Colonies of a range of sizes are possible to observe in Sentinel-1, from the biggest colony on the continent (Coulman Island) with a population of 19,822, at last count^44^, to Astrid, which is observable at least in some years, with a population of just 1368^32^ at last count. While we recommend IW mode for its significantly higher resolution (10 m), we show that colonies are also sometimes detectable in the lower-resolution (40 m) EW mode (Fig. S6), which could be of use in cases where IW mode images are not available. HH or HV channels, which detect returning backscatter differently, can both be used for detection (Fig. S7), but the latter was rarely available in IW mode for the colonies we investigated.

We note that complex ice conditions can make colonies more difficult or impossible to locate, particularly if there is a lot of rough or multi-year ice with high backscatter. This is because, as noted above, C-band SAR is sensitive to rough ice and micro-topography (e.g. brine wicking, frost flowers)^52^. Such conditions made it impossible to locate the colony at Snow Hill in some preliminary observations and more difficult in some years at Atka Bay (Fig. S2). Near-range incidence angles (<35°) can make this particularly difficult, as penguin backscatter varies less consistently with incidence angle (Fig. S8) than the background ice, which typically appears brighter at lower angles (Fig. S9). Near-range incidence angles have previously been shown to be more sensitive to sea ice roughness in first-year ice during winter^47, 51^, which forms the vast majority of ice in the study regions. In years where it was more difficult to observe birds due to ice conditions, identification was especially reliant on identifying the consistent movement of a similarly sized colony from one SAR image to the other. While backscatter of the background ice can vary between images (due to environmental changes at the surface), only penguins could be responsible for the distinct movement observed on the stationary fast ice, as visible in Videos S1–3. Other issues that made a colony more difficult to identify on fast ice included the penguins being more dispersed or the colony being very close to the margin of the high-backscatter ice shelf.

We also demonstrate that, at times (usually late summer), guano is detectable in Sentinel-1 SAR imagery on ice shelves, as well as fast ice, at Atka Bay (Fig. 6) and SANAE (Fig. S4). Given that the guano is not always evident in the SAR imagery even when visible in optical imagery, and only becomes visible in SAR in late summer (Fig. 6), it is likely that the guano is causing a distinct lowering of backscatter indirectly. While we do not have ground-truth data to determine why this might be the case, we hypothesise that the SAR imagery is detecting increased melt caused by the low albedo of the guano, and/or algae and other impurities associated with colonies^50^. For example, Sentinel-1 is known to be able to detect meltwater on ice shelves, due to absorption of the signal by wet snow (e.g. ref. ^49^). Although summer observations of guano on ice shelves are already possible using optical imagery (e.g. refs. ^13, 43^), the ability to detect guano on ice shelves in SAR could prove useful for filling temporal or spatial gaps where optical images are not available during summer.

The visibility of emperor penguins in Sentinel-1 suggests that other SAR instruments may also be of use, such as TerraSAR-X that was reported as having potential^42^. Sentinel-1 has the advantage of being freely available and having a relatively high spatial and temporal coverage. However, higher-resolution missions could reveal details not possible in Sentinel-1, including the movements of smaller sub-colony groups. Furthermore, in the context of understanding colony movement and response to local environmental conditions, it is important to investigate whether and how they are observable in different wavelengths, such as X-band and L-band, and how they can be better distinguished from the background backscatter of fast ice or ice shelves. In particular, opportunities may become available via the newly launched NASA-ISRO NISAR mission, which started collecting L-band and S-band images over the Antarctic coast in late 2025. High-resolution SAR images in June to July would be particularly valuable for assessing breeding populations, as this is when the least variation in numbers is expected^6^.

### Colony movements

We demonstrate the potential for detailed year-round emperor penguin tracking using a combination of multiple optical instruments and Sentinel-1 SAR. In doing so, we observe several key behaviours at the Atka Bay, Coulman Island and Cape Washington colonies.

Our remote sensing observations capture the movement of colonies during winter and show that colonies remained in a relatively stable location during the winter compared to later in the summer. Typically, they remained within ~0.50 km of their earliest observed location throughout the winter (Fig. 9). Colonies remained in a broadly similar location throughout the stages of the winter breeding cycle, including egg-laying and the return of the females (Videos S1–3). However, some movement takes place through the winter, with movement detectable between virtually every successive SAR image, e.g. colonies moving from sheltering directly in the lee of the ice shelf at Atka Bay to more exposed areas on the sea ice (Figs. 3–5). Of all years and study sites, the Atka Bay colony in 2022 was most mobile during winter. In that year, by 7 July the colony had moved over 1.50 km from its first location on 17 April, and up to the neighbouring small embayment to the north, albeit for reasons that are not yet obvious (Video S4f).

Following a relatively stationary period during winter, the colonies moved further away in ~September–December (Fig. 9) when both parents must forage simultaneously, and chicks are preparing to fledge^4, 6^. There were examples at all three colonies of at least one group reaching over 5 km from their initial early-winter location (Fig. S5). Notably, movement was most variable between years at Atka Bay, while there was significant variation in the timing of increased mobility between the three colonies. While the Cape Washington colony was relatively stationary (not exceeding 1 km from the first-winter location) until becoming more mobile in late October–November, at Coulman Island there was increasing mobility from July, then more so in September, and at Atka Bay the spike in mobility ranged from May to November.

As summer progresses, colonies become increasingly dominated by chicks. Although they must move closer to the fast ice edge, or at least tidal cracks, to eventually head out to sea, we show that all three colonies move away from open water. For example, the colony at Atka Bay typically moved onto the ice shelf and towards its interior, away from the fast ice (Fig. 8 and Video S4). We observed the colony moving from the fast ice onto the ice shelf in seven of the 8 years in our study period (Fig. 8 and Video S4), and the colony consistently moved onto the ice shelf regardless of any apparent change in stability in the fast ice. Fast ice did not break out early (i.e. before the end of December) at Atka Bay in any of our study years, and the colony made this transition months before breakout. We show that the vast majority of the colony spent winter on the fast ice, only moving onto the ice shelf in the spring, and most often in September. At Coulman Island (Fig. 10 and Video S5) the colony typically first moved towards the ice edge before turning away, while at Cape Washington (Video S6) they tended to move away from the fast ice edge in late summer. At Coulman Island and Cape Washington, the penguins may opt to enter the water through tide cracks rather than at the fast ice edge, but at Atka Bay there is unlikely to be an entry point to the sea from the interior of the ice shelf.

In addition to our observations, previous studies have observed multiple colonies moving onto ice shelves, including the Atka Bay colony^13, 41, 53, 54^. It has been debated whether movement of colonies away from the ice edge, particularly onto ice shelves, is an adaptation to thinning ice conditions or a response to other local environmental factors and meteorological conditions^41, 54^. Our observations of colonies moving away from the ice edge, including onto the ice shelf, during periods of stable fast ice, and of multiple groups within a colony changing direction simultaneously and coherently, support field evidence that wind changes in speed and direction, rather than ice conditions, may be the main driver of such movements^41, 55^. As Antarctic sea ice may have entered a new state^14, 16^ that could potentially reduce fast ice stability^18^, the environmental/climatic drivers of colony movement could become more complicated, and ice shelves could play an increasingly important role as habitat for emperor penguins. Consequently, greater knowledge and understanding of colony movements in relation to the fast ice edge, and particularly onto ice shelves, is needed to better assess and model colonies’ vulnerability to future conditions. As climate reanalysis data are generally not suitable (due to spatial resolution) for analysing the relationship between penguin movement and meteorological factors, which can be very local and rapidly changing^41^, further studies using high spatial and temporal resolution meteorological data collected in situ, and including both wind speed and direction data, would improve understanding of these processes.

### Implications and applications of year-round monitoring

Improved temporal observations, combined with better understanding of why colony groups move as they do, will be important in the context of future protection of the species. In particular, improved knowledge of the winter range of colonies may help establish well-defined exclusion zones around colonies, as has been suggested as part of proposals to support emperor penguins if they were to become a specially protected species^56^. Furthermore, determining how best to protect emperor penguins may be reliant on having winter observations in order to understand how colonies respond to rapidly evolving winter sea ice conditions^14–16^, including potential fast ice changes in response to reduced regional sea ice extents^18^ such as late fast ice formation impacting the arrival time and location of returning penguins. Additionally, changes in the scale and frequency of glacier calving events may cause winter sea ice breakout events and other disruptions that impact penguins^13, 21, 22^. As we demonstrate, the response to these changes and disruptions could be analysed using SAR. Moreover, Sentinel-1 could also possibly be used to fill gaps in colony observations during summer, particularly when optical images are not collected or are not available due to cloud cover. Thus, by enabling year-round multi-platform observations of colony movements and their sea ice and glaciological setting, not only will behaviour be better understood, but packages of protection can be developed.

We demonstrate the potential for near-live monitoring throughout the year, using free, medium-resolution optical and SAR imagery. This includes monitoring the movement of colony sub-groups in summer at sub-weekly resolution. Such monitoring done regularly and at scale would clearly benefit from automated methods. Thus, the observations presented in this paper provide a useful dataset to develop and test a more physically grounded, statistically validated, automated detection framework that can reliably discriminate the penguins from the complexities of sea ice, using radiometric thresholds, textural analysis and change-detection. Although complicated in SAR by ice features with high backscatter and differing incidence angles between images, and in optical imagery by the complexity of changes in guano coverage, automation is likely feasible with developments in machine learning alongside extensive training data. Development of such an approach would also benefit from an investigation of the physical explanation for the backscatter response to penguins, possibly requiring fieldwork. While beyond the scope of this study, by demonstrating the potential to track colonies manually using the recently expanded suite of instruments, we aim to stimulate further study using advanced automated methods.

## Conclusions

We demonstrate that Sentinel-1 SAR can be used to observe movement of emperor penguin colonies throughout the winter by remote sensing. By combining winter SAR imagery with summer optical imagery, we are able to track the movement of colonies year-round, including at the level of sub-groups. We find that the Atka Bay, Coulman Island and Cape Washington colonies are all less mobile during winter compared to summer, but there is variation between years and colonies. All colonies tend to move away from the fast ice edge at times (including towards the ice shelf interior at Atka Bay), despite its apparent stability, and coherent movement across groups within a colony is suggestive of meteorological drivers dominating. Our work demonstrates the potential of multi-sensor remote sensing, including SAR, as an important tool in understanding emperor penguin behaviour throughout their entire breeding cycle. Moreover, it highlights SAR’s potential to inform decision-making about the species’ vulnerability in a warming climate.

## Methods

Sentinel-1 and other C-band SAR instruments have been used extensively to study a variety of sea ice conditions (e.g. refs. ^45–47^), but not for identification of penguins. SAR backscatter is sensitive to many properties of the surface it interacts with, such as roughness, moisture content and salinity. Although these relationships are better understood in the Arctic and can be complicated in the Antarctic by seawater flooding of sea ice, winter first-year ice typically has a relatively low and constant backscatter. This is due to the signal penetrating to the snow-ice interface where it scatters from the brine layer^52, 57^. There is a positive correlation between roughness and backscatter, particularly at lower incidence angles, from the centimetre scale to large metre-plus features such as blocks of ice. Older multiyear ice also appears brighter due to penetration of the signal beyond the surface into the ice and high volume scattering^47, 52^. As described, we found that penguin colonies also cause high backscatter returns that are perceptible on winter fast ice.

In contrast to first-year sea ice, dry ice shelves cause relatively high backscatter returns due to volume scattering from the firn and higher roughness. Melt can be detected on ice shelves using Sentinel-1 SAR because the moisture causes increased absorption and reflection away from the instrument^49^. As described, we find that penguin guano can sometimes be detected, which we assume to be due to its influence on melt.

### Sentinel-1 imagery processing

Sentinel-1 SAR was inspected in Google Earth Engine (GEE). This allowed us to easily cycle between and analyse vast amounts of imagery (391 images at Cape Washington, 328 at Coulman Island, 444 at Atka Bay) without the challenges of processing and storing large quantities of data. Google Earth Engine applies a series of pre-processing steps to Sentinel-1 images (https://developers.google.com/earth-engine/guides/sentinel1, last accessed: 20 August 2025): (1) application of orbit file, (2) GRD border noise removal, (3) thermal noise removal, (4) radiometric calibration and (5) orthorectification. Images are also converted to decibels (dB). Backscatter values for colonies and the surrounding fast ice were obtained in GEE, using a script to draw polygons around the feature of interest and obtain the mean and maximum backscatter within the polygon.

The timelapse movies presented as Videos S1–3 were produced using the European Space Agency (ESA)’s Copernicus platform (https://browser.dataspace.copernicus.eu/). These, as well as screenshots of images in GEE, were used to identify colonies by their movement in the SAR. Screenshots from GEE provided more flexibility than the timelapse videos in terms of adjusting zoom level and focusing on areas of interest. All available images from 1 April to 31 August (or later) were analysed, for both instruments Sentinel-1A and -1B and all tracks that covered the area of interest. As Sentinel-1 is not impacted by cloud cover, this includes all images that were captured. Our study period covers the years 2017–2024. Some images were available in prior years, since the launch of satellite A on 3 April 2014, but images are more plentiful from 2017 when both satellites A and B became available. 

For figures presented in the paper, Sentinel-1 images were downloaded from the Alaska Satellite Facility (https://search.asf.alaska.edu/) and processed in ESA’s SNAP toolbox. Images were calibrated, had thermal noise removed, projected and converted to decibels.

### Backscatter values and Sentinel-1 modes and channels

Sentinel-1’s IW mode was used for identifying colonies because of its higher resolution (10 m) compared to the extra wide (EW) mode (40 m). In addition, the horizontal-horizontal (HH) channel was used rather than the horizontal-vertical (HV) because the satellite only collected HH images in the vast majority of cases. Figures 3–5 show the mean and maximum backscatter of the colony pixels, with the lower mean values typically being due to more low-backscatter fast ice (non-colony) pixels being measured, due to the colony being spread out in a cluster. In a sample year for each colony, the winter mean of the calculated mean backscatter within a sample area of fast ice was −16.2 dB (Atka Bay), −18.7 dB (Coulman Island) and −18.2 dB (Cape Washington) (Fig. S9a–c), and the mean maximum fast ice backscatter was −9.6 dB (Atka Bay), −12.2 dB (Coulman Island) and −11.5 dB (Cape Washington) (Fig. S9d–f). In contrast, the winter mean of mean backscatter for colony pixels was higher, at −8.9 dB (Atka Bay), −9.4 dB (Coulman Island) and −10.9 dB (Cape Washington) (Fig. S8a–c) and the winter mean of maximum colony backscatter was −4.9 dB (Atka Bay), −5.3 dB (Coulman Island) and −4.5 dB (Cape Washington) (Fig. S8d–f). We calculated the ‘signal to clutter’ ratio for the Atka Bay data (due to the fact it was easier to separate the signal here), and found a mean of 8.1 dB, with values ranging from 4.5 to 11.4 dB (Table S1). Notably, the mean backscatter of the fast ice was substantially higher at near-range incidence angles (<35°), while incidence angle had little impact on the backscatter of colony pixels (Figs. S8 and S9). This means that the contrast between colony pixels and fast ice pixels was stronger at medium-far range incidence angles (>35°), making the colony easier to identify.

Two examples of where images were captured in both the HH and HV channels of the IW mode were analysed at Coulman Island and Cape Washington (none were found at Atka Bay). Colonies were also evident in the HV images, with both the fast ice and colonies having lower backscatter (Fig. S7). In an image from Coulman Island on 27 April 2017, the colony had a mean backscatter of −11.1 dB and maximum backscatter of −6.4 dB in the HH channel, and −19.6 and −12.1 dB, respectively, in the HV channel. Similarly, at Cape Washington on 15 May 2016, the colony had a mean of −13.8 dB and a maximum of −5.2 dB in HH, and −23.8 dB and a maximum of −7.1 dB in HV. Thus, although less often available than the HH channel, the HV channel also proved viable for identifying colonies.

Examples of the lower-resolution EW mode were also analysed in HH mode. Colonies were more difficult to spot in these 40 m resolution images, but could sometimes be distinguished. In two example images at Atka Bay on 8 May and 31 July 2023, the colony could clearly be spotted (Fig. S6a, b). While it could likely be spotted on 23 August 2020 in EW mode at Coulman Island too, in a 31 July 2020 image the colony could not be discerned in EW mode despite being visible in the IW mode during this period (Fig. S6c, d). Overall, although less useful than IW mode due to issues of spatial resolution, the EW mode proved viable in some cases for identifying colonies.

Guano on the ice shelf during summer often covered a much larger area than is typical for a colony on fast ice in winter, and was easily identifiable in EW mode, in both the HH channel and especially the HV channel where the backscatter was lower (Fig. S4).

### Colony tracking

Colony tracking was done manually in GEE using an adapted version of the Google Earth Engine Digitisation Tool (GEEDiT)^58^. A semi-automated approach was tested, based on thresholding of backscatter values to identify high-backscatter areas, and manual exclusion of icebergs and rough ice (see Supplementary Materials). However, although the approach worked well in some scenarios, a manual method was found to perform better overall due to the semi-automated approach’s limitations in areas/years of extensive high-backscatter ice. The semi-automated approach, tested with multiple different backscatter thresholds, generated substantial false positives that then required a large degree of manual intervention. While we acknowledge that a small amount of human error could have occurred and automated approaches may have benefits in terms of a standardised approach that is easy to reproduce, manual approaches often perform better when feasible to carry out, and are common in cryosphere^59–61^ and penguin science, particularly in the complex environment of sea ice in SAR imagery^62^. Numerous penguin studies visually identify penguin colonies in optical imagery (e.g. refs. ^2, 12, 13, 34^), and when automated or supervised approaches are used, visual identification is used as the validation data (e.g. refs. ^26, 27, 31, 33^). Although optical imagery is different to SAR, this highlights the value of visual interpretation as an approach. Additionally, previous automated approaches using optical imagery^31, 33^, while useful for some applications, generated substantial false positives, and were focused on detecting all guano, rather than strictly penguins, which negates their utility for the goals of this study in the spring/summer period.

Sentinel-1 SAR IW mode images in the HH channel (10 m resolution) were used from the first image in which the colony was identifiable after 1 April, until the colony was first visible in optical imagery (September). It was done for all years 2017–2024 at the three study sites. The optical platforms Landsat 8, Landsat 9 and Sentinel-2 were all used during the summer, with Sentinel-2 preferred to Landsat when images from multiple platforms were available on the same day, due to Sentinel-2’s higher spatial resolution (10 m vs 30 m). Optical images were displayed with the near-infrared, green and blue bands mapped to R, G, B, respectively due to guano’s high reflectivity at near-infrared wavelengths (e.g. refs. ^34^).

During winter, a point was placed at the location(s) of the colony based on visual inspection of the Sentinel-1 image. If the colony appeared to be in more than one distinct cluster, multiple points were placed. At Atka Bay, the colony was visible as 1–2 cohesive shapes (Fig. 3) and thus determining the number of groups was simple. At Coulman Island and Cape Washington, the colonies were often more diffuse (Figs. 4 and 5) and classifying the number of groups was less clear. In those cases, we classified a cluster of bright penguin pixels as a group if they seemed to be largely connected and moving together. A higher resolution instrument would be necessary to accurately determine the number of small groups and huddles. In this paper, we attempted to capture the broad dynamics of the colonies as best as possible. At times a colony was not necessarily clearly distinct from other high backscatter features (e.g. rough ice, small icebergs and multiyear ice) in a static image, but viewing a timelapse of images enabled confident identification of the colony from the distinct and consistent movement of the colony in contrast to the stable fast ice (including high-backscatter icebergs and rough ice). The colony moving close to the high backscatter ice shelf (at Atka Bay) could also make finding the colony challenging in a single image, but, again, it was possible to identify by observing movement. To estimate the uncertainty associated with colony location point placement, we quantified the area of the colony cluster/grouping (i.e. the grouping within which each point was located) using a sample of winter images (2017 and 2024) from each colony. Colony area was estimated for 30 images (Table S2), yielding a mean of 14,422 m^2^ across all colonies, with site-specific means of 5450 m^2^ (Atka Bay), 13,790 m^2^ (Coulman Island) and 28,520 m^2^ (Cape Washington). Changes in incidence angle when using more than one Sentinel-1 track through a season could lead to an offset of approximately 1–3 pixels (10 m pixel spacing) of estimated colony location.

From the first appearance of optical imagery after the return of sufficient sunlight to the study sites, tracking was done in optical imagery. In these medium-resolution optical images, it was broadly guano rather than penguins that was directly observed. Penguins would be present within the guano stain, but there would also be areas of guano that were remnant and therefore represented evidence of recent past penguin presence. However, due to the high temporal resolution of optical images (sub-weekly, at times daily) in recent years, especially when combining three platforms, it was possible to reasonably infer penguin presence from movement/spread of the guano and the colour of the guano e.g. Fig. 7. A recent study^2^ noted that penguin presence can be distinguished from remnant guano in Sentinel-2. If new guano appeared since the previous image, we inferred that penguins had moved there. When the guano spread in different directions, then the penguins were assumed to be moving as separate groups within the colony, and we assigned a point to each group. Although it was not always possible to discern if there were also penguins at ‘old’ stain sites, often it was clearly only remnant staining because it faded without renewal, or alternatively it continued to darken, suggesting there was still a group there. When there was a large guano stain (e.g. >200 m across; such as often develops at Cape Washington around their original location) that appeared to currently have penguins (due to renewing staining), determining whether to classify it as one group or multiple connected groups could be difficult, and we based this on whether there appeared to be multiple ‘cores’ or a mostly even spread around one core. Towards the end of summer, the guano stains tended to become fainter, as the population reduced, eventually making the colony undetectable.

As in the SAR, the spatial resolution of optical imagery limited detection of small groups of penguins that cause small and minimal guano staining. Indeed, occasionally particularly small groups were detectable in Sentinel-2 that were not detectable in Landsat. Additionally, there will have been instances of penguin movement that were missed where penguins remained within the bounds of old staining and caused imperceptible or barely perceptible changes in its colour. However, through manual inspection of changes in guano, we were able to infer broad and key changes in the colony’s location.

We note that in our analysis we found evidence of guano staining on an ice shelf that was buried by snowfall and then became visible again the following year, as was previously observed by Macdonald et al.^13^. Guano that formed in 2023–24 was visible late in the summer of 2024–25 (Fig. S10). It was clear that it was old staining rather than new, as it did not spread from the current year’s guano and it formed an identical pattern to the previous year. Furthermore, the guano all gradually became visible at once (as its snow cover was removed), rather than spreading across the ice. As the staining was clearly old, we do not consider it to be a significant source of error here, but the appearance of old guano should be considered when using guano as a way to detect penguins.

## Supplementary information

## Data Availability

Supplementary data includes shapefiles for all colony tracking. These data are available at the UK Polar Data Centre: https://doi.org/10.5285/e1e00e5d-fc4c-4948-9e00-8d02e8b359d9.
