# Supplementary material for: Year-round movement of emperor penguin colonies observed from space
Source: Commun Earth Environ. 2026 Sep 2;7(1):703. doi: 10.1038/s43247-026-03906-0 (PMC13538044; doi:10.1038/s43247-026-03906-0)
Supplement: Supplementary file 1 [file 43247_2026_3906_MOESM51_ESM.pdf]

nature portfolio

Peer Review File

Year-round movement of emperor penguin colonies observed from space

Corresponding Author: Dr Grant Macdonald

This file contains all reviewer reports in order by version, followed by all author rebuttals in order by version.

Version 0:

Reviewer comments:

Reviewer #1

(Remarks to the Author)
While the manuscript "Sentinel-1 Synthetic Aperture Radar (SAR) Enables Year-Round Remote Sensing Observations of Emperor Penguins" addresses a compelling gap in ecological monitoring—the "polar night blind spot"—I find that the current study does not meet the high standards of methodological rigor and quantitative validation required for publication. The study is largely descriptive and phenomenological, lacking the robust physical interpretation and statistical validation necessary to establish this approach as a reliable scientific tool. My primary concerns focus on the methodology and the validation analysis:

1. Methodological Subjectivity and Lack of Automation
A fundamental weakness of the study is its reliance on manual visual interpretation ("visual inspection" and "manual tracking") to identify colony locations in noisy SAR imagery. In the context of modern remote sensing, particularly for a journal of this caliber, a methodology based on subjective human interpretation of unquantified signal contrasts is insufficient. The authors acknowledge that identifying colonies is difficult in complex ice conditions (e.g., rough ice, ridges), yet they do not provide a quantitative signal-to-clutter ratio analysis or a reproducible, semi-automated detection algorithm. Without a defined radiometric threshold or a texture-based classification model, the method is difficult to scale or reproduce objectively. The distinction between a "penguin colony" and "deformed sea ice" currently rests on the observer's intuition regarding movement patterns, which introduces significant confirmation bias.

2. Ambiguity in Physical Scattering Mechanisms
The manuscript fails to provide a convincing physical explanation for the observed SAR backscatter. The authors speculate that the high backscatter (dB) is due to surface roughness or dielectric properties of the birds, but this remains a qualitative hypothesis without modeling support. C-band SAR (5.6 cm wavelength) is highly sensitive to sea ice micro-topography (brine wicking, frost flowers, cm-scale roughness). The study does not demonstrate how the biophysical signal of a penguin huddle is radiometrically distinct from the background variability of winter sea ice, which also exhibits high backscatter variability due to deformation and age. The "guano melt" hypothesis on ice shelves is similarly speculative. Without separating the biogenic contribution to the radar cross-section from the glaciological background via polarimetric decomposition or rigorous statistical distribution analysis, the "detection" remains ambiguous.

3. Insufficient Validation and "Ground Truth"
The validation strategy is temporally disjointed and logically circular. The authors validate their winter SAR observations (April–August) primarily by linking them to summer optical imagery (September–January). However, this leaves the critical winter period—where the core novelty of the paper lies—without direct validation ("ground truth"). The assumption that a moving high-backscatter target in winter must be a penguin colony because it spatially aligns with a summer optical target is plausible but unproven. There is a non-negligible risk that the tracked features in winter could, at times, be rafting sea ice or icebergs moving with the same ocean currents that influence the penguins. Furthermore, the ecological conclusion that colony movement is driven by wind is presented as an assertion rather than a result derived from quantitative covariance analysis between colony displacement vectors and meteorological data.

Conclusion
In summary, while the observational potential is intriguing, the study in its current form represents a preliminary feasibility

demonstration rather than a fully developed, validated remote sensing methodology. To be considered for publication, the work would need to move beyond manual tracking to demonstrate a physically grounded, statistically validated, and preferably automated detection framework that can reliably discriminate biological targets from complex sea ice clutter. I recommend that this work be submitted to a more specialized journal focused on polar biology or applied remote sensing.

Reviewer #2

(Remarks to the Author)
I have completed the review of the manuscript "Sentinel-1 Synthetic Aperture Radar (SAR) Enables Year-Round Remote Sensing Observations of Emperor Penguins", whose topic I found interesting and relevant due to its methodological value and its potential to overcome some of the limitations in observing Emperor Penguin colonies during the austral winter. I would like to congratulate the authors on their good work. The manuscript is written in a concise, well-structured, and clear manner, making it easy to follow the line of argument and to understand what was done, how it was done, and the importance of the study in filling an observational gap for the first time using remote sensing. The figures are of high quality and contain all the necessary elements to aid the reader's interpretation alongside the manuscript content. As a result, I recommend acceptance of the manuscript for publication. I only have a few brief comments, suggestions, or questions that arose after reading the manuscript.

- Line 118. Could you please add a reference for the Coulman Island penguin census?

- Results section. The section is well explained, and the results are clearly presented. Nevertheless, I encourage the authors to make an effort to integrate some figures and reduce the total number of figures in the manuscript, which is currently quite large. I suggest the following grouping, although it is ultimately the authors' decision how to proceed:
a) Fig. 2;
b) Fig. 3 + Fig. 4 + Fig. 5;
c) Fig. 6;
d) Fig. 7 + Fig. 8 + Fig. 10;
e) Fig. 9.

- Lines 301–303. I agree with the authors that the winter SAR signal seems to correspond more closely to colony movement than to the guano stain left behind; however, this appears to somewhat contradict what is shown in Figure 6. Could the authors elaborate a bit more on this point?

- Lines 514–516. Why was machine learning not used instead of manually tracking the colonies?

Reviewer #3

(Remarks to the Author)
I read the manuscript with great interest. The authors present the use of freely available Sentinel-1 SAR data (primarily IW mode, HH polarisation) to deliver the first year-round satellite observations of emperor penguin (*Aptenodytes forsteri*) colonies during the Antarctic polar winter, when conventional optical remote sensing is effectively unavailable. They demonstrate that colonies can be detected in winter on land-fast sea ice (fast ice) as areas of elevated backscatter relative to the surrounding fast ice. A key element of the approach is manual colony tracking in SAR time series, where distinguishing colonies from sea-ice features is facilitated by the consistent movement of the colony signal against a comparatively static fast-ice background. The analysis combines winter SAR observations (April/May–September) with summer optical observations (Sentinel-2 and Landsat 8/9; September–January), enabling reconstruction of continuous seasonal time series of colony positions and sub-group locations across the full breeding cycle. This approach enables detailed multi-year tracking of three colonies (Atka Bay, Coulman Island, and Cape Washington) over 2017–2024, often at sub-weekly temporal resolution; allows assessment of seasonal mobility, showing limited winter movement and increased spatial dynamics from late winter/spring into summer; captures within-season changes in colony structure, including frequent splitting into multiple groups and movements of several kilometres; documents repeatable habitat transitions, notably the regular spring shift at Atka Bay from fast ice to the adjacent ice shelf despite apparently stable fast-ice conditions; and shows that late-summer guano-associated areas on ice shelves can sometimes be detected in Sentinel-1 as zones of reduced backscatter, plausibly linked indirectly to melt processes and/or impurities (e.g., algae) altering snow/firn properties.

Overall, the study highlights how integrating SAR and optical data enables high-frequency monitoring of colonies on year-round timescales, addressing a major observational gap in colony behaviour during the polar night and providing a basis for developing automated approaches to colony detection and tracking under rapidly changing Antarctic sea-ice conditions. The study is well motivated and transparently described, and the figures and text are well prepared. I did not identify methodological flaws or substantive issues that would affect the conclusions, and overall I consider this a high-quality, genuinely innovative contribution of broad interest to both the cryosphere and ecological remote sensing communities. Beyond its scientific contribution, this work is relevant to science-to-policy initiatives such as Ant-ICON (Antarctic–Integrated Science to Inform Conservation), SOOS (Southern Ocean Observing System), and CCAMLR (Commission for the Conservation of Antarctic Marine Living Resources), which share an interest in advancing innovative, non-invasive, long-term, and large-area observation methods to support conservation, environmental management, and evidence-based decision-making in Antarctica and the Southern Ocean.

Therefore, I recommend that the editors accept the manuscript pending minor revision.

My comments and suggestions for improvement focus on a few minor aspects. The manuscript appropriately acknowledges that manual colony identification can be challenging under complex fast-ice conditions and varying incidence angles, and that detection may at times rely on temporal consistency across successive SAR scenes. As a minor enhancement, the authors could consider formalising this by reporting a simple measure of repeatability and/or uncertainty for the manual tracking (e.g., agreement between two independent annotators for a subset of scenes, and/or a typical positional uncertainty expressed in pixels/metres, optionally accompanied by a qualitative confidence category). This would further increase transparency and strengthen the robustness and transferability of the proposed workflow.

I have no concerns regarding the reference list, which reflects the authors' extensive and long-standing expertise in this research area; the relatively frequent self-citation appears appropriate given their sustained contributions over many years. As a minor literature/context addition, the authors may wish to consider citing Mustafa et al. (2012), which discusses satellite-based monitoring of Antarctic *Pygoscelis* penguin colonies and includes radar-related components, to better contextualise the statement that SAR has not been used to detect penguins (or to clarify the distinction between earlier radar use and the present Sentinel-1 winter application). Massom et al. (2009) could also be cited as key background on fast-ice variability and implications for emperor penguins.

Suggested references:
Mustafa, O., Pfeifer, C., Peter, H.-U., Kopp, M., & Metzig, R. (2012). Pilot study on monitoring climate-induced changes in penguin colonies in the Antarctic using satellite images (TEXTE 19/2012; Report No. UBA-FB 001601/E; Project No. FKZ 3711 85 199). Federal Environment Agency (Umweltbundesamt).
Massom, R. A., Hill, K., Barbraud, C., Adams, N., Ancel, A., Emmerson, L., & Pook, M. J. (2009). Fast Ice Distribution in Adélie Land, East Antarctica: Interannual Variability and Implications for Emperor Penguins (Aptenodytes forsteri).Marine Ecology Progress Series, 374, 243–257. https://doi.org/10.3354/meps07734

The Results section is very informative; however, I would suggest considering whether some of the narrative descriptions of colony movements could be slightly condensed. For example, the main patterns (winter stability vs. increased spring/summer mobility) could be summarised in the main text using a few key ranges or summary statistics, with more detailed year-by-year examples and distances moved to the Supplementary Information and/or figure captions. This may improve readability without weakening the results.

I also suggest considering the placement of the contextual paragraph beginning (lines: 130–135), "Sentinel-1 and other C-band Synthetic Aperture Radar (SAR) instruments are sensitive to a range of sea ice characteristics, such as …". This section reads primarily as background/contextual framing (capabilities of C-band SAR, prior applications to sea ice and glaciers, and the statement that SAR has not been used to detect penguins). Moving all or part of this paragraph to the Discussion (or possibly the end of the Introduction) would help keep the Results focused on the empirical findings. This is a minor structural adjustment, but it may strengthen the flow of the manuscript and the separation between results and interpretation/context.

Finally, there is a minor punctuation issue: in the sentence "During winter, a point was placed at the location(s) of the colony based on visual inspection of the Sentinel-1 image If the colony appeared to be in more than one distinct group, multiple points were placed." a full stop appears to be missing after "image" (i.e., "…Sentinel-1 image. If the colony appeared…").

Overall, the figures and text are well prepared, the writing is clear, and the formatting aligns well with the scope and standards of Communications Earth & Environment.

Version 1:

Reviewer comments:

Reviewer #2

(Remarks to the Author)
The authors have addressed the suggested changes and have clearly responded to the questions raised. I still believe that there are too many figures and that some of them could perhaps be moved to the Supplementary Material or combined. Nevertheless, it is up to the editor to make that decision. Otherwise, I recommend the publication of the manuscript once the comments from the other reviewers have been addressed.

Reviewer #3

(Remarks to the Author)
I would like to thank the authors for their careful revisions and detailed responses. All of my previous comments have been satisfactorily addressed, and I have no further concerns regarding the issues I raised.

I consider this study to be an important and timely demonstration of the potential of Sentinel-1 SAR for monitoring emperor penguins year-round, particularly in terms of addressing the observational gap during the polar night. While the approach is partly qualitative and relies on expert interpretation, this does not invalidate the core observational findings.

I therefore support publication, provided that the exploratory nature of the study and its methodological limitations are clearly stated. The manuscript should be presented as a proof of concept, paving the way for the future development of more quantitative and automated approaches.

In my opinion, the novelty and potential impact of the study justify publication at this stage.

Reviewer #4

(Remarks to the Author)
This paper uses Sentinel-1 SAR to detect the movement characteristics of the emperor penguin colonies, which is a very valuable research. However, the current article is more focused on the research report, and the current methods mostly rely on manual interpretation and multi-temporal movement features. Is this methodology applicable in other contexts? The authors are hoped to adopt quantitative machine learning models such as the random forest method to achieve this, so as to improve the reusability of the paper.
(1) Extract the polarization and texture features of the emperor penguin colonies and the background ice. Sentinel-1 provides dual polarization (VV + VH or HH + HV) in the IW mode, and the authors mostly used the HH feature, sometimes also the HV feature. Polarization ratios (HH/HV or VV/VH), polarization differences, polarization entropy, etc. can be used. Since the emperor penguin colonies are high-density discrete points forming patches in space, they are different from the continuous linear textures of ice ridges, so some texture features can be constructed, such as extracting the contrast, correlation, energy, homogeneity, etc. of the polarization data. It is suggested that the authors can quantitatively express the features of the emperor penguin colonies and the ice using the above indicators.
(2) The construction of the measured sample data is the key to model building. It is recommended that the authors build a sample database of the emperor penguin colonies. For example, the emperor penguin colony samples can be set as "1", while the samples of rough background ice, multi-year ice, small icebergs, ice ridges, etc. can be set as "0". In addition, the authors have manually drawn the positions of the colonies over many years, which can exactly serve as the labels for the training samples. The authors currently rely mainly on optical images from August to September for validation, but the coverage of the "1" sample in other time periods has no optical verification. This is also a problem, and it is necessary to have verified optical data so that the "1" sample is truly valid.
(3) With the polarization features and texture features extracted in (1), in (2), select the labels to train the random forest model or XGBOOST model. I think this method has comparability and certain repeatability.
(4) Finally, the model verification is recommended. It is suggested to use the center points of the emperor penguin colonies as the reference true value and calculate the Euclidean distance error of the colony center points detected by the above (3) model.

Open Access This Peer Review File is licensed under a Creative Commons Attribution 4.0 International License, which permits use, sharing, adaptation, distribution and reproduction in any medium or format, as long as you give appropriate credit to the original author(s) and the source, provide a link to the Creative Commons license, and indicate if changes were made.
In cases where reviewers are anonymous, credit should be given to 'Anonymous Referee' and the source.
The images or other third party material in this Peer Review File are included in the article’s Creative Commons license, unless indicated otherwise in a credit line to the material. If material is not included in the article’s Creative Commons license and your intended use is not permitted by statutory regulation or exceeds the permitted use, you will need to obtain permission directly from the copyright holder.

To view a copy of this license, visit https://creativecommons.org/licenses/by/4.0/

Dear Dr Marisa McDonald and three anonymous reviewers,

Thank you for taking the time to review our paper and for the valuable comments. We submit a revised version which we believe is substantially improved thanks to changes made in response to the suggestions of the reviewers. Below we include the reviewer comments and our response (in blue text) to each remark.

Best regards,

Dr Grant Macdonald, on behalf of all authors

Reviewer #1 (Remarks to the Author):

While the manuscript "Sentinel-1 Synthetic Aperture Radar (SAR) Enables Year-Round Remote Sensing Observations of Emperor Penguins" addresses a compelling gap in ecological monitoring—the "polar night blind spot"—I find that the current study does not meet the high standards of methodological rigor and quantitative validation required for publication. The study is largely descriptive and phenomenological, lacking the robust physical interpretation and statistical validation necessary to establish this approach as a reliable scientific tool. My primary concerns focus on the methodology and the validation analysis:

We thank the reviewer for their comments and for acknowledging our effort to address a compelling gap in ecological monitoring during the 'polar blind spot'. This is a first attempt at learning how to detect emperor penguins during winter with Sentinel-1 SAR, and we agree that further steps should be taken to attempt to develop an automated, reproducible approach. To address this feedback, we have included a new section about our experimentation with a semi-automated approach, where we discuss the difficulties in employing such an approach. We find that manual, visual interpretation performs better for a number of reasons, but we discuss recommended next steps for the community regarding the use of SAR for penguin detection. We also include new quantitative data to support the assessment of uncertainty in our SAR tracking, new data comparing signal and clutter, a new optical validation figure, and other changes to text.

1. Methodological Subjectivity and Lack of Automation

A fundamental weakness of the study is its reliance on manual visual interpretation ("visual inspection" and "manual tracking") to identify colony locations in noisy SAR imagery. In the context of modern remote sensing, particularly for a journal of this caliber, a methodology based on subjective human interpretation of unquantified signal contrasts is insufficient. The authors acknowledge that identifying colonies is difficult in complex ice conditions (e.g., rough ice, ridges), yet they do not provide a quantitative signal-to-clutter ratio analysis or a reproducible, semi-automated detection algorithm. Without a defined radiometric threshold or a texture-based classification model, the

method is difficult to scale or reproduce objectively. The distinction between a "penguin colony" and "deformed sea ice" currently rests on the observer's intuition regarding movement patterns, which introduces significant confirmation bias.

As noted, we appreciate the reviewer's concerns and have carefully considered this issue. We also acknowledge that our rationale/justification for using a manual approach could be clearer and have now addressed this comment in the revised manuscript (lines **539-554** – all line numbers refer to the 'clean' version). In response, we wish to make three important points: **(1)** In our testing, semi-automated methods do not work as well as visual interpretation (especially interpretation of pattern in movement); **(2)** Manual mapping based on human expertise is not inherently less reliable than automated/semi-automated methods, and is commonly used in remote sensing studies of the cryosphere and of penguins; **(3)** Our cross validation of SAR images with optical data includes the winter SAR tracking period (April-September). These three points are explained in more detail below, and are made more explicitly in the text.

More specifically:

(1) We have tested a semi-automated detection approach based on thresholding of backscatter values for penguins in Sentinel-1 imagery. We now include this in the supplementary material to help the reader compare its effectiveness with manual mapping (sub-section titled *'An investigation of semi-automated emperor penguin detection in winter Sentinel-1 SAR*). While we find that the approach works quite well in some scenarios, the results are variable and, overall, it is clear that a manual approach is more reliable for the colonies in our study. The semi-automated approach identifies high-backscatter pixels. However, these include not only penguin pixels, but also icebergs, and areas of rough or older ice (bright ice). The icebergs and bright ice then need to be excluded by manual intervention. Where there is limited bright ice in the study area, such as in the example of Coulman Island in 2024 (Fig. S11), the approach can be quite effectively used for tracking penguins. However, in other examples, such as Cape Washington in 2024 (Fig. S12) or Atka Bay in 2021 (Fig. S13), the amount of bright ice in the study area is such that the degree of manual-intervention necessary to exclude these pixels makes the approach less effective than the manual approach we used in this paper. The semi-automated approach therefore becomes much more time-consuming than the manual approach, and the high degree of manual intervention makes it no more 'objective'. Crucially, where the semi-automated approach does work, we note that it does not result in any significant change in the results we presented in the paper.

Nonetheless, there may be potential for developing an automated approach for tracking penguins in SAR (e.g. more sophisticated change-detection and machine-learning, including the use of a substantial amount of training data). This is mentioned

on lines **435-442** of the manuscript. We also include the code and illustrative figures for our tested semi-automated approach in the supplementary material to support further experimentation in the community.

(2) Automated methods have benefits in terms of a standardised approach that is quick and easy to apply to multiple areas and we encourage their development, but they do not necessarily achieve better results. For example, in terms of SAR's use in a sea ice environment, Kortum et al. (2024, pg. 1) noted "*even with the inception of advanced machine learning methods and modern data analysis, a universally reliable classifier does not yet exist to retrieve sea ice classes from [synthetic aperture] radar imagery...[and] human generated ice charts are still dominant in operation usage*".

Visual interpretation (as opposed to automated analysis) of optical imagery is also a commonly used approach in the fields of glaciology, sea ice research and monitoring, and remote sensing of penguins (e.g. Leeson et al., 2013; Withialahowsky et al., 2025; Wienecke et al., 2024; Fretwell, 2026). Indeed, there are several papers that note that manual mapping is more reliable in various fields. Efforts to automate penguin guano detection in optical imagery (e.g. Shen et al., 2017; Liu et al., 2025) use visual interpretation as validation data and report substantial false positives that that would preclude a tracking study of this nature. Thus, although optical imagery is different from SAR imagery, this nevertheless highlights the value of visual interpretation as an approach that has been the foundation of many of the significant advances in understanding the presence and vulnerability of emperor penguins in the last decade. We justify this in the text on lines **539-554**, but also acknowledge the potential for human error.

To address the reviewer comment, we have also carried out new analysis to quantify uncertainty in our manual interpretation of SAR, detailed on lines **576-580** and summarized in the new Table S2. We also note that the paper already includes supplementary figures that quantify the backscatter of the penguins in comparison to the background fast ice (Fig. S8-9), providing quantitative information on the signal contrast and a key objective element used in the visual interpretation. Additionally, the new Table S1 shares 'signal to clutter ratio' data.

(3) We do cross-validate examples of SAR imagery with near-simultaneous optical imagery during our winter tracking period (which runs April-September) (Fig. 2), and now add an additional August example (Fig. S3). In addition to the characteristic movement of the penguins on stationary ice (which is visible over multiple SAR images), these optical images provide confidence that penguins are being observed.

2. Ambiguity in Physical Scattering Mechanisms

The manuscript fails to provide a convincing physical explanation for the observed SAR backscatter. The authors speculate that the high backscatter (dB) is due to surface

roughness or dielectric properties of the birds, but this remains a qualitative hypothesis without modeling support. C-band SAR (5.6 cm wavelength) is highly sensitive to sea ice micro-topography (brine wicking, frost flowers, cm-scale roughness). The study does not demonstrate how the biophysical signal of a penguin huddle is radiometrically distinct from the background variability of winter sea ice, which also exhibits high backscatter variability due to deformation and age. The "guano melt" hypothesis on ice shelves is similarly speculative. Without separating the biogenic contribution to the radar cross-section from the glaciological background via polarimetric decomposition or rigorous statistical distribution analysis, the "detection" remains ambiguous.

We acknowledge that sea ice features can also cause high backscatter for a variety of reasons, and we acknowledge that we were not sufficiently clear about this issue. However, no ice features move around on top of stationary ice in the characteristic fashion that penguins do. This movement is visible in the animations (e.g. Video S1a). We address this in new comments on lines **327-329** and **334-338**.

Modelling to prove what mechanism is responsible for the high backscatter of the penguins would be beyond the scope of the study, but we thought it valuable to offer some plausible speculation. Standing at >1 m tall, the logic of our ‘penguin roughness’ hypothesis is eminently plausible because such scales of roughness are routinely detectable by SAR imagery. We are also confident in our ‘guano melt’ hypothesis (guano decreases surface albedo and can therefore change the near-surface structure of the snowpack and thus alter backscatter), but we acknowledge we cannot directly test it. We agree investigations of the mechanism would be valuable but suggest that this would be beyond the scope of this initial study. To be clearer, and to help guide future work we now add text to the manuscript based on the reviewers comments at lines **309-312**, **344-345, and 440-442.**

Importantly, we highlight that the mechanism responsible for their high backscatter does not impact the key results of the paper.

3. Insufficient Validation and "Ground Truth"

The validation strategy is temporally disjointed and logically circular. The authors validate their winter SAR observations (April–August) primarily by linking them to summer optical imagery (September–January). However, this leaves the critical winter period—where the core novelty of the paper lies—without direct validation ("ground truth"). The assumption that a moving high-backscatter target in winter must be a penguin colony because it spatially aligns with a summer optical target is plausible but unproven. There is a non-negligible risk that the tracked features in winter could, at times, be rafting sea ice or icebergs moving with the same ocean currents that influence the penguins. Furthermore, the ecological conclusion that colony movement is driven by wind is presented as an assertion rather than a result derived from

quantitative covariance analysis between colony displacement vectors and meteorological data.

We agree that we were not sufficiently clear about our validation and its limitations. Our 'winter' tracking period runs from late April to September, and we validate with optical images in September (Fig. 2) i.e. within that period. These optical/SAR validation pairs are captured on the same day or one day apart, and we are unclear why this is a logically circular validation. Additionally, we now add a new example from August (Fig. S3). The inclusion of earlier optical images (May-July) would be ideal, but unfortunately they are simply not available due to the lack of daylight. We add text to lines **147-150**.

Additionally, while there can be some low-magnitude 'flickering' of bright areas of ice due to changes in incidence angle or small environmental changes that impact backscatter, it is nevertheless evident that the fast ice and icebergs are positionally and structurally stable, in contrast to the colony which moves around on the surface in a distinct manner i.e. there is no risk that we could be mistaking penguins for moving icebergs or rafting ice. For this reason, as mentioned above, it may not always be clear from a single SAR image where a colony is but its location then becomes clear by using a sequence of images in close succession. We try to illustrate this in Figs. 3-5 as best as possible in fixed images, but this is also why we provide supplementary videos (Videos S1-S3) of the SAR imagery through a season. In combination with the optical validation that shows penguins exhibit high backscatter (Figs. 2; S3), we can be confident that penguins are being observed.

We also now note that a non-peer reviewed report by the German Space Agency (DLR) reported detection of emperor penguins in TerraSARX imagery in 2012, though without examples or any further analysis (this report by Mustafa et al. (2012) is now cited in the manuscript – lines **86-88**). We acknowledge in the paper there are instances where detection is difficult or impossible, but this does not discredit the many instances where penguins *are* detectable, just as the difficulty of locating some colonies in optical imagery does not negate the value of that key approach (for example, Fretwell (2026), in this journal, states "*at first arrival, penguins groups…were difficult to distinguish from other environmental features but…")*.

We acknowledge that the discussions regarding the reason for colony movement are based on evidence around the sea ice conditions and discussion of previous findings in the literature, rather than quantitative meteorological data. Unfortunately, meteorological data at the temporal and spatial resolution necessary to carry out such an assessment is not available to carry out this analysis. For example, while there is a meteorological station at Neumayer research station in the vicinity of Atka Bay colony, crucially there is no wind direction data available for the study period. We agree such an analysis would be valuable. We add text to the manuscript at lines **410-414**.

Conclusion

In summary, while the observational potential is intriguing, the study in its current form represents a preliminary feasibility demonstration rather than a fully developed, validated remote sensing methodology. To be considered for publication, the work would need to move beyond manual tracking to demonstrate a physically grounded, statistically validated, and preferably automated detection framework that can reliably discriminate biological targets from complex sea ice clutter. I recommend that this work be submitted to a more specialized journal focused on polar biology or applied remote sensing.

Thank you again for the review, which we believe has helped strengthen the paper. We hope that our responses and changes offer some reassurance, and we reiterate three key points: (1) Semi-automated methods do not work as well as visual interpretation for this study; (2) Manual mapping based on human expertise is commonly used in remote sensing studies of the cryosphere and penguins, and has proved superior here and in other studies, leading to many of the major advances in the remote sensing of emperor penguins; (3) Our period of cross-validation of SAR with optical imagery includes part of our winter analysis period. The review has helpfully prompted us to include a **new supplementary section detailing the testing of a semi-automated approach**, **new data (Table S2) for assessing the confidence of the location during SAR tracking**, **an additional validation figure (Fig. S3)** and **additional quantitative data on the 'signal' versus 'clutter' backscatter (Table S1,** accompanying Figs. S8-9**)**. Additionally, numerous other text additions/changes have been made.

Reviewer #2 (Remarks to the Author):

I have completed the review of the manuscript "Sentinel-1 Synthetic Aperture Radar (SAR) Enables Year-Round Remote Sensing Observations of Emperor Penguins", whose topic I found interesting and relevant due to its methodological value and its potential to overcome some of the limitations in observing Emperor Penguin colonies during the austral winter. I would like to congratulate the authors on their good work. The manuscript is written in a concise, well-structured, and clear manner, making it easy to follow the line of argument and to understand what was done, how it was done, and the importance of the study in filling an observational gap for the first time using remote sensing. The figures are of high quality and contain all the necessary elements to aid the reader's interpretation alongside the manuscript content. As a result, I recommend acceptance of the manuscript for publication. I only have a few brief comments, suggestions, or questions that arose after reading the manuscript.

Thank you for your review.

- Line 118. Could you please add a reference for the Coulman Island penguin census?

The Coulman Island number shares the same reference as the Cape Washington number that follows. To avoid any ambiguity we now repeat that reference after the Coulman Island number as well.

- Results section. The section is well explained, and the results are clearly presented. Nevertheless, I encourage the authors to make an effort to integrate some figures and reduce the total number of figures in the manuscript, which is currently quite large. I suggest the following grouping, although it is ultimately the authors' decision how to proceed:

a) Fig. 2;

b) Fig. 3 + Fig. 4 + Fig. 5;

c) Fig. 6;

d) Fig. 7 + Fig. 8 + Fig. 10;

e) Fig. 9.

We are open to making these changes but fear that combining them would make them too small in the publication. We have not made this change, but will do so if the editor requires it.

- Lines 301–303. I agree with the authors that the winter SAR signal seems to correspond more closely to colony movement than to the guano stain left behind; however, this appears to somewhat contradict what is shown in Figure 6. Could the authors elaborate a bit more on this point?

Note that this is the case on *fast ice,* where the penguins have *high* backscatter. Figure 6 shows how guano presents in the SAR and optical imagery on the *ice shelf* in summer, where the there is *low* backscatter in the SAR signal at times, likely due to increased melt where there is guano.

This is discussed in the sub-section 'Identification of guano on ice shelves using SAR' and at lines **340-350** in the Discussion.

- Lines 514–516. Why was machine learning not used instead of manually tracking the colonies?

We tested a semi-automated approach for the SAR imagery that we now discuss in the supplementary material, but we found that a manual approach was more appropriate (see full response to Review #1). We believe a machine learning approach could have potential in developing an automated tracking system and have now acknowledged/discussed this in the revised manuscript, but that would require substantially more training data and is beyond the scope of this study of their winter movement.

Reviewer #3 (Remarks to the Author):

I read the manuscript with great interest. The authors present the use of freely available Sentinel-1 SAR data (primarily IW mode, HH polarisation) to deliver the first year-round satellite observations of emperor penguin (Aptenodytes forsteri) colonies during the Antarctic polar winter, when conventional optical remote sensing is effectively unavailable. They demonstrate that colonies can be detected in winter on land-fast sea ice (fast ice) as areas of elevated backscatter relative to the surrounding fast ice. A key element of the approach is manual colony tracking in SAR time series, where distinguishing colonies from sea-ice features is facilitated by the consistent movement of the colony signal against a comparatively static fast-ice background. The analysis combines winter SAR observations (April/May–September) with summer optical observations (Sentinel-2 and Landsat 8/9; September–January), enabling reconstruction of continuous seasonal time series of colony positions and sub-group locations across the full breeding cycle. This approach enables detailed multi-year tracking of three colonies (Atka Bay, Coulman Island, and Cape Washington) over 2017–2024, often at sub-weekly temporal resolution; allows assessment of seasonal mobility, showing limited winter movement and increased spatial dynamics from late winter/spring into summer; captures within-season changes in colony structure, including frequent splitting into multiple groups and movements of several kilometres; documents repeatable habitat transitions, notably the regular spring shift at Atka Bay from fast ice to the adjacent ice shelf despite apparently stable fast-ice conditions; and shows that late-summer guano-associated areas on ice shelves can sometimes be detected in Sentinel-1 as zones of reduced backscatter, plausibly linked indirectly to melt processes and/or impurities (e.g., algae) altering snow/firn properties.

Overall, the study highlights how integrating SAR and optical data enables high-frequency monitoring of colonies on year-round timescales, addressing a major observational gap in colony behaviour during the polar night and providing a basis for developing automated approaches to colony detection and tracking under rapidly changing Antarctic sea-ice conditions. The study is well motivated and transparently described, and the figures and text are well prepared. I did not identify methodological flaws or substantive issues that would affect the conclusions, and overall I consider this a high-quality, genuinely innovative contribution of broad interest to both the cryosphere and ecological remote sensing communities. Beyond its scientific contribution, this work is relevant to science-to-policy initiatives such as Ant-ICON (Antarctic–Integrated Science to Inform Conservation), SOOS (Southern Ocean Observing System), and CCAMLR (Commission for the Conservation of Antarctic Marine Living Resources), which share an interest in advancing innovative, non-invasive, long-term, and large-area observation methods to support conservation, environmental management, and evidence-based decision-making in Antarctica and the Southern Ocean.

Therefore, I recommend that the editors accept the manuscript pending minor revision.

Thank you for your review and positive comments.

My comments and suggestions for improvement focus on a few minor aspects. The manuscript appropriately acknowledges that manual colony identification can be challenging under complex fast-ice conditions and varying incidence angles, and that detection may at times rely on temporal consistency across successive SAR scenes. As a minor enhancement, the authors could consider formalising this by reporting a simple measure of repeatability and/or uncertainty for the manual tracking (e.g., agreement between two independent annotators for a subset of scenes, and/or a typical positional uncertainty expressed in pixels/metres, optionally accompanied by a qualitative confidence category). This would further increase transparency and strengthen the robustness and transferability of the proposed workflow.

We have made new measurements and added a new table to provide some uncertainty data for the placement of points when tracking in colonies in SAR. We estimated the area of the colony grouping in thirty SAR scenes (ten at each colony, across two winters), in which a tracking point was placed. This is summarised in Table S2 and referred to on lines **576-580**.

I have no concerns regarding the reference list, which reflects the authors' extensive and long-standing expertise in this research area; the relatively frequent self-citation appears appropriate given their sustained contributions over many years. As a minor literature/context addition, the authors may wish to consider citing Mustafa et al. (2012), which discusses satellite-based monitoring of Antarctic Pygoscelis penguin colonies and includes radar-related components, to better contextualise the statement that SAR has not been used to detect penguins (or to clarify the distinction between earlier radar use and the present Sentinel-1 winter application). Massom et al. (2009) could also be cited as key background on fast-ice variability and implications for emperor penguins.

Suggested references:

Mustafa, O., Pfeifer, C., Peter, H.-U., Kopp, M., & Metzig, R. (2012). Pilot study on monitoring climate-induced changes in penguin colonies in the Antarctic using satellite images (TEXTE 19/2012; Report No. UBA-FB 001601/E; Project No. FKZ 3711 85 199). Federal Environment Agency (Umweltbundesamt).

Massom, R. A., Hill, K., Barbraud, C., Adams, N., Ancel, A., Emmerson, L., & Pook, M. J. (2009). Fast Ice Distribution in Adélie Land, East Antarctica: Interannual Variability and Implications for Emperor Penguins (Aptenodytes forsteri).Marine Ecology Progress Series, 374, 243–257.
https://doi.org/10.3354/meps07734

Thank you for the suggestions, we have now added both of these references. We have also added text explaining that there was a previous observation in TerraSAR-X by Mustafa et al. (**lines 86-88**).

The Results section is very informative; however, I would suggest considering whether some of the narrative descriptions of colony movements could be slightly condensed. For example, the main patterns (winter stability vs. increased spring/summer mobility) could be summarised in the main text using a few key ranges or summary statistics, with more detailed year-by-year examples and distances moved to the Supplementary Information and/or figure captions. This may improve readability without weakening the results.

We feel the paper is already on the shorter side, and the narrative description for each of the three colonies is only 220-400 words for the whole study period. We would prefer to not move any of this to the supplementary material, but we are willing to do so if the editor prefers.

I also suggest considering the placement of the contextual paragraph beginning (lines: 130–135), "Sentinel-1 and other C-band Synthetic Aperture Radar (SAR) instruments are sensitive to a range of sea ice characteristics, such as …". This section reads primarily as background/contextual framing (capabilities of C-band SAR, prior applications to sea ice and glaciers, and the statement that SAR has not been used to detect penguins). Moving all or part of this paragraph to the Discussion (or possibly the end of the Introduction) would help keep the Results focused on the empirical findings. This is a minor structural adjustment, but it may strengthen the flow of the manuscript and the separation between results and interpretation/context.

As suggested, we have moved this to the first paragraph to the Discussion.

Finally, there is a minor punctuation issue: in the sentence "During winter, a point was placed at the location(s) of the colony based on visual inspection of the Sentinel-1 image If the colony appeared to be in more than one distinct group, multiple points were placed." a full stop appears to be missing after "image" (i.e., "...Sentinel-1 image. If the colony appeared...").

We have amended this.

Overall, the figures and text are well prepared, the writing is clear, and the formatting aligns well with the scope and standards of Communications Earth & Environment.

Dear Dr Marisa McDonald and three anonymous reviewers,

Thank you for taking the time to review our paper again. We address the reviews below in blue text.

Best regards,

Dr Grant Macdonald, on behalf of all authors

Reviewer #2 (Remarks to the Author):

The authors have addressed the suggested changes and have clearly responded to the questions raised. I still believe that there are too many figures and that some of them could perhaps be moved to the Supplementary Material or combined. Nevertheless, it is up to the editor to make that decision. Otherwise, I recommend the publication of the manuscript once the comments from the other reviewers have been addressed.

Thank you for the review and positive comments. As before, we prefer to keep the figures in the main text, and think that combining them could make them harder to follow, but are happy to move to supplementary material if the editor requests it.

Reviewer #3 (Remarks to the Author):

I would like to thank the authors for their careful revisions and detailed responses. All of my previous comments have been satisfactorily addressed, and I have no further concerns regarding the issues I raised.

I consider this study to be an important and timely demonstration of the potential of Sentinel-1 SAR for monitoring emperor penguins year-round, particularly in terms of addressing the observational gap during the polar night. While the approach is partly qualitative and relies on expert interpretation, this does not invalidate the core observational findings.

I therefore support publication, provided that the exploratory nature of the study and its methodological limitations are clearly stated. The manuscript should be presented as a proof of concept, paving the way for the future development of more quantitative and automated approaches.

In my opinion, the novelty and potential impact of the study justify publication at this stage.

Thank you for the review and positive comments.

Reviewer #4 (Remarks to the Author):

This paper uses Sentinel-1 SAR to detect the movement characteristics of the emperor penguin colonies, which is a very valuable research. However, the current article is more focused on the research report, and the current methods mostly rely on manual interpretation and multi-temporal movement features. Is this methodology applicable in other contexts? The authors are hoped to adopt quantitative machine learning models such as the random forest method to achieve this, so as to improve the reusability of the paper.
(1) Extract the polarization and texture features of the emperor penguin colonies and the background ice. Sentinel-1 provides dual polarization (VV + VH or HH + HV) in the IW mode, and the authors mostly used the HH feature, sometimes also the HV feature. Polarization ratios (HH/HV or VV/VH), polarization differences, polarization entropy, etc. can be used. Since the emperor penguin colonies are high-density discrete points forming patches in space, they are different from the continuous linear textures of ice ridges, so some texture features can be constructed, such as extracting the contrast, correlation, energy, homogeneity, etc. of the polarization data. It is suggested that the authors can quantitatively express the features of the emperor penguin colonies and the ice using the above indicators.
(2) The construction of the measured sample data is the key to model building. It is recommended that the authors build a sample database of the emperor penguin colonies. For example, the emperor penguin colony samples can be set as "1", while the samples of rough background ice, multi-year ice, small icebergs, ice ridges, etc. can be set as "0". In addition, the authors have manually drawn the positions of the colonies over many years, which can exactly serve as the labels for the training samples. The authors currently rely mainly on optical images from August to September for validation, but the coverage of the "1" sample in other time periods has no optical verification. This is also a problem, and it is necessary to have verified optical data so that the "1" sample is truly valid.
(3) With the polarization features and texture features extracted in (1), in (2), select the labels to train the random forest model or XGBOOST model. I think this method has comparability and certain repeatability.
(4) Finally, the model verification is recommended. It is suggested to use the center points of the emperor penguin colonies as the reference true value and calculate the Euclidean distance error of the colony center points detected by the above (3) model.

Thank you for your review and we should state at the outset that we are largely in agreement with the sentiments of the reviewer regarding the Methods. However, this is not the main focus of this particular manuscript. As the reviewer mentions, the manuscript largely focusses on the ‘research report’. This is intentional because the step-change presented in this work is the discovery of the ability to monitor, using freely available data, the year-round movement of emperor penguin colonies.

We agree with the reviewer that an automated approach to tracking emperor penguin colonies in Sentinel-1 imagery would be very valuable, and that a method such as random forest could potentially be effective. However, we believe that development of an automated approach warrants a thorough and systematic analysis of multiple automated/semi-automated methods that should be written up as a separate ‘methods-testing’ paper, including change-detection, random forest and other classifiers, as well as a full validation procedure. This will allow us to undertake

the additional work to collect a large amount of training data and then test a variety of methods to determine the pros and cons of each approach and make recommendations for best practice. In fact, this is exactly what we are planning to do in our future work that is currently written in to a funding proposal (decision pending).

Importantly, we also wish to highlight that implementing an automated approach at this stage would change the focus of the paper substantially but would not alter the key scientific findings of this ‘discovery’ paper regarding the year-round observations of emperor penguins.

For both of the above reasons (i.e. the scale of the technical analysis required and the minimal impact it would have on the key science finding of this paper) we choose not to alter the methodological approach applied in this particular manuscript. We also note that this decision is also guided by reviewers 1 and 2 who indicate they would accept the manuscript without further changes to the methodological approach.
