# Supplementary material for: Year-round movement of emperor penguin colonies observed from space
Source: Commun Earth Environ. 2026 Sep 2;7(1):703. doi: 10.1038/s43247-026-03906-0 (PMC13538044; doi:10.1038/s43247-026-03906-0)
Supplement: Supplementary file 2 [file 43247_2026_3906_MOESM1_ESM.docx]

**SUPPLEMENTARY MATERIAL**

*An investigation of semi-automated emperor penguin detection in winter Sentinel-1 SAR*

A semi-automated approach for identifying the location of emperor penguin colonies in Sentinel-1 SAR during winter was tested but ultimately not used for analysis because it was found that manual detection was better suited for the study. This was primarily due to the semi-automated approach often identifying a large number of false positives that required a very large degree of manual intervention. Nevertheless, we include a description of the semi-automated approach, which showed promise in some scenarios.

The method was based on thresholding of backscatter values alongside manual intervention to exclude icebergs and other non-penguin high-backscatter pixels (such as rough and multiyear ice). The method was applied in Google Earth Engine to Sentinel-1 IW mode HH images, as was used for manual tracking. First, a region of interest polygon was applied to limit the analysis to the relevant fast ice area and exclude the adjacent land (Coulman Island and Cape Washington) or ice shelf (Atka Bay). Second, we aimed to manually exclude icebergs and areas of high backscatter ice by applying polygons that excluded these features. Third, a backscatter threshold was applied, with groups of pixels above that threshold identified as penguins. As discussed in the methods and shown in Figs. S8-9, penguin pixels have a relatively high backscatter but are not uniform in value. Multiple thresholds were tested, and a set of examples for each colony shows thresholds set at -11 dB, -9 dB, and -8 dB (Figs. S11-S13). Lower thresholds capture more of the pixels occupied by the penguins but identify more false positives (ice).

In some test cases, this approach worked quite well and only required limited manual intervention for application, showing potential for use in the tracking analysis. This was the case at Coulman Island in 2024, as shown in Fig. S11, where a cluster of penguins was identified and only two icebergs and one area of high backscatter ice had to be excluded (although movement of the penguins into this excluded region would cause problems with the approach). However, in other cases, such as the examples at Cape Washington in 2024 (Fig. S12) and Atka Bay in 2021 (Fig. S13), high-backscatter pixels were spread throughout the region of interest, to the extent that the level of manual intervention needed for excluding these pixels was more time-consuming than a fully-manual approach, and negated the advantages of semi-automation over a manual approach (reproducibility, scaling, and objectivity). We suggest that an automated approach would likely also require a threshold that varies by incidence angle, and possibly by time-of-year and configuration of the penguins too (e.g. spread out or densely packed).

While there are advantages to a semi-automated approach, such as an identification based on an objective backscatter value and better reproducibility, the fact that the approach only functioned well in some scenarios led us to choose a manual approach that performed better and could be applied to all sites and years at the scale of this study. This is partly the case because, in addition to identifying backscatter values, movement plays a key role in the identification of the penguins. Preliminary testing suggests that automated or semi-automated approaches have potential, but would likely require the use of change-detection and sophisticated machine-learning methods trained with substantial amounts of training data, beyond the scope of this study.

The code for our preliminary semi-automated approach is available at: *https://code.earthengine.google.com/ec29ba9e5e8f7d4bfb6ee6ffdea90030*

**SUPPLEMENTARY FIGURES**

**Fig. S1:** Examples of penguins visible in Sentinel-1 SAR IW HH images during winter at the (a) SANAE, (b) Mertz, and (c) Astrid colonies. The locations of the colonies are shown in Fig. 1a.

**Fig. S2:** Examples of colonies that were difficult or impossible to identify due to ice conditions. (a) We were unable to identify the Snow Hill colony in this image with complex ice conditions, despite an optical image from the same day showing their presence in the circled area; (b) In the winter of 2020, an influx of rough, older ice made the Atka Bay colony more difficult to identify, particularly in a single image as above. However, by observing a timelapse of imagery and focusing on the movement of the colony, we were able to identify them.

**Fig. S3**: Penguin guano and colonies in near-simultaneous optical and SAR imagery. (a) A Sentinel-2 optical image and (b) Sentinel-1 SAR image at the same location at the SANAE colony one day apart. Yellow arrows point to bright areas showing the colony, where dark guano indicative of current penguin presence can also be seen in the same area in the optical imagery.

**Fig. S4:** Evidence of guano from the SANAE colony on the Trolltunga ice tongue (ice shelf) in optical imagery and different Sentinel-1 modes. The guano is visible (a) on the ice shelf on 23 January in a Sentinel-2 optical image and on 15 January in (b) an IW mode HH image, (c) EW mode HV image and (d) EW mode HH image. The backscatter of the guano was estimated for all Sentinel-1 images.

**Fig. S5:** Maximum distance travelled throughout a year by subgroups at the Atka Bay, Coulman Island and Cape Washington colonies for 2017-2024. The maximum distance of all groups of the colony on each day is calculated from the earliest location of the colony in that year. In the instance (2024) where there are two separate groups at Atka Bay throughout winter in different bays, the distance is calculated for the southern of the two groups.

**Fig. S6:** Examples of colony visibility in lower-resolution (40 m) EW mode (HH channel) imagery. (a-b) The Atka Bay colony is less clear than in IW mode but still discernible on 8 May and 31 July 2023. The images show the same region as in Fig. 3; (c) We are unable to discern the Coulman Island colony on 31 July 2020 (d) but can do so on 23 August 2020. (c-d) show the same region as in Fig. 4. As with IW mode, observing the movement of the colonies aids in identification.

**Fig. S7:** Comparisons of colonies in IW HV and HH imagery, where images were captured simultaneously in both channels. HV and HH images of the (a-b) Coulman Island colony on 27 April 2017 and (c-d) Cape Washington on 15 May 2016. An image was used from before the study period (2017-2024) as very few IW HV images were captured. Note the different colour scaling for HV as backscatter values are lower. The mean and maximum backscatter were calculated for all images for an approximate area of the colony’s location.

(a)

(b)

(c)

(d)

(e)

(f)

**Fig. S8:** Mean and maximum colony Sentinel-1 backscatter (dB) for a sample winter at each colony. The mean and maximum backscatter were calculated for pixels identified as being part of the colony, although, particularly when the colony is more diffuse, pixels that do not contain penguins have likely been included. The incidence angle (0 d.p.) of each image is also displayed. (a-c) Mean backscatter for Atka Bay, Coulman Island, and Cape Washington colony pixels, respectively; (d-f) Maximum backscatter for Atka Bay, Coulman Island, and Cape Washington colony pixels, respectively.

(a)

(b)

(c)

(d)

(e)

(f)

**Fig. S9:** Mean and maximum fast ice Sentinel-1 backscatter (dB) for a sample winter at each colony. The mean and maximum backscatter were calculated for a patch of fast ice in the vicinity of each colony that did not contain the colony. The incidence angle (0 d.p.) of each image is also displayed. (a-c) Mean backscatter for Atka Bay, Coulman Island, and Cape Washington fast ice, respectively; (d-f) Maximum backscatter for Atka Bay, Coulman Island, and Cape Washington fast ice, respectively.

**Fig. S10:** An example of guano from a previous year becoming exposed and visible on the ice shelf at Atka Bay. A Sentinel-2 image from 8 February 2024 shows guano from the current year. An image from the following breeding season on 23 January 2025 shows that, in addition to new guano, the previous year’s guano is faintly visible, evident from the same pattern.

**Fig. S11:** Attempted (semi-)automated detection of penguins in Sentinel-1 using thresholding of backscatter values at Coulman Island. Three different threshold values (a: -11 dB, b: -9 dB, c: -8 dB) are displayed for the same image of the colony on 28^th^ April 2024. The location of the penguins, as known from analysing both the individual image and a timelapse of imagery, is shown by the arrow. While the lower threshold captures more pixels where the penguins are present, it also captures substantially more high-backscatter ice. In these examples, we found a semi-automated approach was feasible and reasonably practical, as 1-2 limited ‘exclusion polygons’ could be applied to exclude high-backscatter ice, as well as the icebergs.

**Fig. S12:** Attempted (semi-)automated detection of penguins in Sentinel-1 using thresholding of backscatter values at Cape Washington. Three different threshold values (a: -11 dB, b: -9 dB, c: -8 dB) are displayed for the same image of the colony on 22^nd^ July 2024. The location of the penguins, as known from analysing both the individual image and a timelapse of imagery, is shown by the arrow. In these examples, we found a semi-automated approach was not practical in comparison with manual interpretation and tracking, as a large degree of manual intervention was necessary to exclude false positives from high-backscatter ice and to check results.

**Fig. S13:** Attempted (semi-)automated detection of penguins in Sentinel-1 using thresholding of backscatter values at Atka Bay. Three different threshold values (a: -11 dB, b: -9 dB, c: -8 dB) are displayed for the same image of the colony on 7^th^ May 2021. The location of the penguins, as known from analysing both the individual image and a timelapse of imagery, is shown by the arrow. Viewing the motion played a particularly important role in this example with a large amount of high-backscatter ice. In these examples, we found a semi-automated approach was not practical in comparison with manual interpretation and tracking, because an unfeasibly large degree of manual intervention would be necessary to exclude false positives from high-backscatter ice and to check results against visual interpretation.

| Date | Penguin backscatter (dB) | Fast ice backscatter (dB) | Signal-to-clutter ratio (dB) |
| --- | --- | --- | --- |
| 06/05/2023 | -9.61 | -16.15 | 6.54 |
| 07/05/2023 | -5.41 | -16.77 | 11.36 |
| 15/05/2023 | -9.59 | -14.48 | 4.89 |
| 18/05/2023 | -10.47 | -16.08 | 5.61 |
| 19/05/2023 | -8.80 | -16.97 | 8.17 |
| 27/05/2023 | -10.12 | -14.84 | 4.72 |
| 30/05/2023 | -8.00 | -16.32 | 8.32 |
| 31/05/2023 | -7.67 | -17.12 | 9.45 |
| 08/06/2023 | -10.44 | -14.98 | 4.54 |
| 11/06/2023 | -10.79 | -16.72 | 5.93 |
| 12/06/2023 | -7.36 | -17.36 | 10.00 |
| 20/06/2023 | -5.74 | -15.27 | 9.53 |
| 23/06/2023 | -9.06 | -16.83 | 7.77 |
| 24/06/2023 | -6.95 | -17.58 | 10.63 |
| 02/07/2023 | -7.01 | -15.06 | 8.05 |
| 05/07/2023 | -11.94 | -17.36 | 5.42 |
| 06/07/2023 | -10.68 | -17.98 | 7.30 |
| 14/07/2023 | -8.71 | -15.21 | 6.50 |
| 17/07/2023 | -8.86 | -16.86 | 8.00 |
| 18/07/2023 | -10.49 | -17.73 | 7.24 |
| 26/07/2023 | -8.12 | -15.78 | 7.66 |
| 29/07/2023 | -8.52 | -17.74 | 9.22 |
| 30/07/2023 | -8.29 | -18.11 | 9.82 |
| 07/08/2023 | -6.21 | -17.03 | 10.82 |
| 10/08/2023 | -9.32 | -18.32 | 9.00 |
| 11/08/2023 | -9.10 | -18.11 | 9.01 |
| 19/08/2023 | -11.65 | -17.47 | 5.82 |
| 22/08/2023 | -9.37 | -18.35 | 8.98 |
| 23/08/2023 | -8.23 | -17.57 | 9.34 |
| 31/08/2023 | -6.22 | -16.99 | 10.77 |
| 03/09/2023 | -6.88 | -17.85 | 10.97 |

**Table S1:** Mean penguin and fast ice backscatter data for Atka Bay in 2023 as presented in Figs. S8a and S9a, and the ‘signal-to-clutter’ ratio, which is calculated by subtracting the ‘clutter’ (fast ice) from the ‘signal’ (penguin) in dB.

| Colony | Date | Area (m^2^) |
| --- | --- | --- |
| Atka Bay | 26/04/2017 | 13100 |
| Atka Bay | 20/05/2017 | 17800 |
| Atka Bay | 24/06/2017 | 5800 |
| Atka Bay | 25/06/2017 | 6500 |
| Atka Bay | 30/07/2017 | 4300 |
| Atka Bay | 31/07/2017 | 3100 |
| Atka Bay | 24/08/2017 | 1700 |
| Atka Bay | 01/05/2024 | 2700 |
| Atka Bay | 24/05/2024 | 1700 |
| Atka Bay | 30/06/2024 | 2800 |
| Atka Bay | 24/07/2024 | 1300 |
| Atka Bay | 29/08/2024 | 4600 |
| Coulman Island | 30/04/2017 | 18500 |
| Coulman Island | 23/05/2017 | 30200 |
| Coulman Island | 29/06/2017 | 13100 |
| Coulman Island | 28/07/2017 | 9300 |
| Coulman Island | 28/08/2017 | 3500 |
| Coulman Island | 29/04/2024 | 16600 |
| Coulman Island | 23/05/2024 | 12500 |
| Coulman Island | 27/06/2024 | 10200 |
| Coulman Island | 22/07/2024 | 7900 |
| Coulman Island | 27/08/2024 | 16100 |
| Cape Washington | 02/05/2017 | 80600 |
| Cape Washington | 26/05/2017 | 86200 |
| Cape Washington | 29/06/2017 | 21700 |
| Cape Washington | 31/07/2017 | 6200 |
| Cape Washington | 30/08/2017 | 14800 |
| Cape Washington | 29/04/2024 | 16100 |
| Cape Washington | 23/05/2024 | 15800 |
| Cape Washington | 30/06/2024 | 9000 |
| Cape Washington | 24/07/2024 | 2900 |
| Cape Washington | 29/08/2024 | 4900 |

**Table S2:** Colony location point-placement uncertainty for a sample of thirty SAR images for all three colonies. The area of the colony grouping was measured for ten images (five in 2017 and five in 2024) at each colony. Points were placed manually around the centre of the grouping. Note pixels are 100 m^2^.
