# Supplementary material for: Year-round movement of emperor penguin colonies observed from space
Source: Commun Earth Environ. 2026 Sep 2;7(1):703. doi: 10.1038/s43247-026-03906-0 (PMC13538044; doi:10.1038/s43247-026-03906-0)
Supplement: Supplementary file 3 [file 43247_2026_3906_MOESM50_ESM.docx]

**Description of Additional Supplementary Files**

**File Name:** Video S1

**Description:**

Time-lapse videos of the Atka Bay colony in Sentinel-1 SAR HH in (a) 2017, (b) 2018, (c) 2019, (d) 2020, (e) 2021, (f) 2022, (g) 2023, (h) 2024.

**File Name:** Video S2

**Description:**

Time-lapse videos of the Coulman Island colony in Sentinel-1 SAR HH in (a) 2017, (b) 2018, (c) 2019, (d) 2020, (e) 2021, (f) 2022, (g) 2023, (h) 2024.

**File Name:** Video S3

**Description:**

Time-lapse videos of the Cape Washington colony in Sentinel-1 SAR HH in (a) 2017, (b) 2018, (c) 2019, (d) 2020, (e) 2021, (f) 2022, (g) 2023, (h) 2024.

**File Name:** Video S4

**Description:**

The Atka Bay colony’s tracked movement throughout (a) 2017, (b) 2018, (c) 2019, (d) 2020, (e) 2021, (f) 2022, (g) 2023, (h) 2024. Dot colour represents the day of year (doy) of colony location, a doy over 365 signifies it is after 1st January of the following year. Note many dates have multiple dots due to splitting of the colony. A single background image is used for each year.

**File Name:** Video S5

**Description:**

The Coulman Island colony’s tracked movement throughout (a) 2017, (b) 2018, (c) 2019, (d) 2020, (e) 2021, (f) 2022, (g) 2023, (h) 2024. Dot colour represents the day of year (doy) of colony location, a doy over 365 signifies it is after 1st January of the following year. Note many dates have multiple dots due to splitting of the colony. A single background image is used for each year.

**File Name:** Video S6

**Description:**

The Cape Washington colony’s tracked movement throughout (a) 2017, (b) 2018, (c) 2019, (d) 2020, (e) 2021, (f) 2022, (g) 2023, (h) 2024. Dot colour represents the day of year (doy) of colony location, a doy over 365 signifies it is after 1st January of the following year. Note many dates have multiple dots due to splitting of the colony. A single background image is used for each year.
